# Time-Resolved Proteome Analysis of *Listeria monocytogenes* during Infection Reveals the Role of the AAA+ Chaperone ClpC for Host Cell Adaptation

**DOI:** 10.1128/mSystems.00215-21

**Published:** 2021-08-03

**Authors:** Marlène S. Birk, Rina Ahmed-Begrich, Stefan Tran, Alexander K. W. Elsholz, Christian K. Frese, Emmanuelle Charpentier

**Affiliations:** a Max Planck Unit for the Science of Pathogens, Berlin, Germany; Weill Cornell Medicine-Qatar

**Keywords:** proteomics, tandem-mass-tag, pulsed SILAC, *Listeria monocytogenes*, proteolysis, ClpC, AAA+, infection, adaptation, host-pathogen interactions, mass spectrometry

## Abstract

The cellular proteome comprises all proteins expressed at a given time and defines an organism’s phenotype under specific growth conditions. The proteome is shaped and remodeled by both protein synthesis and protein degradation. Here, we developed a new method which combines metabolic and chemical isobaric peptide labeling to simultaneously determine the time-resolved protein decay and *de novo* synthesis in an intracellular human pathogen. We showcase this method by investigating the Listeria monocytogenes proteome in the presence and absence of the AAA+ chaperone protein ClpC. ClpC associates with the peptidase ClpP to form an ATP-dependent protease complex and has been shown to play a role in virulence development in L. monocytogenes. However, the mechanism by which ClpC is involved in the survival and proliferation of intracellular L. monocytogenes remains elusive. Employing this new method, we observed extensive proteome remodeling in L. monocytogenes upon interaction with the host, supporting the hypothesis that ClpC-dependent protein degradation is required to initiate bacterial adaptation mechanisms. We identified more than 100 putative ClpC target proteins through their stabilization in a *clpC* deletion strain. Beyond the identification of direct targets, we also observed indirect effects of the *clpC* deletion on the protein abundance in diverse cellular and metabolic pathways, such as iron acquisition and flagellar assembly. Overall, our data highlight the crucial role of ClpC for L. monocytogenes adaptation to the host environment through proteome remodeling.

**IMPORTANCE** Survival and proliferation of pathogenic bacteria inside the host depend on their ability to adapt to the changing environment. Profiling the underlying changes on the bacterial proteome level during the infection process is important to gain a better understanding of the pathogenesis and the host-dependent adaptation processes. The cellular protein abundance is governed by the interplay between protein synthesis and decay. The direct readout of these events during infection can be accomplished using pulsed stable-isotope labeling by amino acids in cell culture (SILAC). Combining this approach with tandem-mass-tag (TMT) labeling enabled multiplexed and time-resolved bacterial proteome quantification during infection. Here, we applied this integrated approach to investigate protein turnover during the temporal progression of adaptation of the human pathogen L. monocytogenes to its host on a system-wide scale. Our experimental approach can easily be transferred to probe the proteome remodeling in other bacteria under a variety of perturbations.

## INTRODUCTION

Listeria monocytogenes is a multisystemic pathogen that causes infections in various host tissues. However, clinical forms of listeriosis show tropism toward the central nervous system and uterus (1), where infections can cause severe symptoms like meningitis and mother-to-fetus infections potentially resulting in miscarriages or still-births (1). For several years, L. monocytogenes has been a model organism for infection because of its ability to invade and multiply within a wide range of phagocytic and nonphagocytic cell lines.

To survive and proliferate in the host during infection, bacterial pathogens must adapt to various hostile changes of the environment by adjusting their proteome’s composition through protein synthesis and degradation (2, 3). Protein degradation is a major determinant to maintain protein homeostasis, and it contributes to the virulence of pathogenic bacteria by controlling the abundance of regulatory proteins and through its involvement in stress response and protein quality control (4–7). ClpC is an Hsp100/Clp protein, an ATP-dependent chaperone that belongs to the AAA+ protein family. ClpC can form an ATP-dependent protease by associating with the peptidase ClpP. Deletion of *clpC* causes L. monocytogenes to be highly vulnerable to multiple stresses, such as iron starvation, higher temperatures (>42°C), and salt stress (8, 9). Moreover, ClpC is required for successful development of virulence (8, 10–12). However, the underlying mechanism by which ClpC is involved in the survival and proliferation of intracellular L. monocytogenes remains elusive.

Mass spectrometry-based proteomics enables the high-throughput and systematic study of proteins on a global scale. In an attempt to characterize the dynamic behavior of human pathogens, several studies have been conducted to analyze proteome changes upon genetic, metabolic, and antibiotic perturbation, most of which were performed *in vitro* (e.g., references 13–16). *In vivo* infection experiments have been carried out to analyze the behavior of pathogens in the context of human host cell-pathogen interactions (e.g., references 17–20); however, these proteomic analyses of intracellular bacteria are primarily built on binary comparisons of the bacterial proteome before and after infection. Therefore, information on accumulation, synthesis, and degradation of proteins is crucial to dissect the regulatory role of protein turnover during pathogenesis. Incorporation of a SILAC label switch upon infection (pulsed SILAC) addresses this limitation and allows the discrimination between proteins synthesized before infection and proteins newly synthesized in response to the host (21, 22). Investigating the dynamic proteome during *in vivo* infection experiments involves a large sample series, necessary for high temporal resolution. Therefore, in this study, we combined pulsed SILAC during infection with TMT multiplexing, allowing high-throughput multiplexed protein quantification (23, 24), to study the temporal progression of L. monocytogenes adaptation to its host. We uncovered extensive remodeling of the wild-type L. monocytogenes proteome in response to the host. Proteins involved in cell proliferation were considerably downregulated, while proteins involved in cellular adaptation to the new environment were substantially more abundant. The *clpC* deletion mutant did not exhibit downregulation of pathways involved in translation to the same extent as the parental strain, indicating the involvement of ClpC in the removal of these proteins. Moreover, our data showed that the *clpC* deletion led to increased synthesis of distinct cellular and metabolic pathways, such as iron acquisition and flagellar assembly. Our approach reveals the pivotal role of ClpC in proteome remodeling required for bacterial adaptation to the host environment.

## RESULTS

### Establishing pulsed SILAC for L. monocytogenes during infection.

To distinguish bacterial proteins synthesized prior to infection from those synthesized in response to the host, we first established metabolic pulse-labeling (pSILAC) for L. monocytogenes. We chose SILAC labeling (25) via stable isotope-coded lysine and constructed a lysine auxotrophic Δ*lysA* mutant to ensure incorporation of the supplied lysine in the medium. We did not observe any significant differences of growth of the Δ*lysA* mutant in a shake flask (*in vitro*) or during infection of Caco-2 cells, a commonly used human epithelial cell model to study host-pathogen interactions, compared to that of the wild type (Fig. S1A, B, and C).

10.1128/mSystems.00215-21.1 FIG S1 Effect of gene deletions on growth and virulence of L. monocytogenes. (A) Growth of the parental strain (EGD-e), the Δ*lysA*Δ*clpC* mutant (Δ*clpC*), and the Δ*lysA* strain (wt) in listeria synthetic medium (LSM). Error bars indicate the standard deviation of the mean. *n* = 3 independent biological experiments. (B) CFU per ml of growth culture comparing the parental strain (EGD-e) with the wt and the Δ*clpC* mutant. Error bars indicate the standard deviation of the mean. *n* = 3 independent biological experiments. (C) Invasion assay based on one time point (4 h post infection). Caco-2 cells were infected with the parental (EGD-e) and the wt strain. Bacteria that were retrieved from the host were plated on BHI agar. CFU per ml were determined to calculate the percentage of cells that successfully entered the host. Error bars indicate the standard deviation of the mean. *n* = 5 independent biological experiments. (D and E) Internalization of the wt strain and the Δ*clpC* mutant. *n* = 3 independent biological experiments. Internalized bacteria were retrieved to determine CFU per ml (D). The percentage of cells that successfully entered the host was calculated. (E) Error bars indicate the standard deviation of the mean. (F) Volcano plot showing differences of protein abundance between the *clpC* deletion mutant (Δ*clpC*) and the wt strain. Red dots indicate proteins significantly more abundant in the Δ*clpC* mutant than in the wt strain. Blue dots indicate proteins significantly less abundant in the Δ*clpC* mutant than in the wt strain (*P*_adj_ ≤ 0.05). *n* = 3 independent biological experiments. Download FIG S1, PDF file, 0.7 MB. Copyright © 2021 Birk et al. 2021 Birk et al. https://creativecommons.org/licenses/by/4.0/ This content is distributed under the terms of the Creative Commons Attribution 4.0 International license.

To investigate the role of ClpC in proteome remodeling during infection, we deleted *clpC* in the Δ*lysA* mutant. Viability of the Δ*lysA*Δ*clpC* mutant was comparable to that of the Δ*lysA* strain and the parental strain (EGD-e) during growth in a shake flask (*in vitro*) (Fig. S1B). However, the Δ*lysA*Δ*clpC* mutant exhibited reduced virulence compared to that of the Δ*lysA* strain (Fig. S1A, D, and E). This is in line with previous studies that reported ClpC involvement in virulence development (8, 11, 12). Since the Δ*lysA* mutant exhibits a wild type-like phenotype, it will be referred to as wt, while the Δ*lysA*Δ*clpC* strain will be referred to as Δ*clpC*.

To characterize the steady-state effect of the *clpC* deletion, we first performed a comprehensive proteome analysis of the wt and Δ*clpC* strains *in vitro*. In total, we detected 25 proteins with significantly higher abundance (fold change [log_2_] of >1, adjusted *P* value [*P*_adj_] of <0.05) in the Δ*clpC* mutant (Fig. S1F and Table S1). This accumulation might be due to either elevated expression levels or a failure to downregulate their abundance through ClpCP protease activity. The dysregulation of these 25 proteins does not appear to influence the growth of the Δ*clpC* mutant *in vitro*; however, their increased abundance could contribute to the decreased adaptability to stress and impaired virulence reported for the Δ*clpC* deletion strain (8–12).

10.1128/mSystems.00215-21.4 TABLE S1 Proteins differentially abundant between the wt and the Δ*clpC* strain. Download Table S1, PDF file, 0.4 MB. Copyright © 2021 Birk et al. 2021 Birk et al. https://creativecommons.org/licenses/by/4.0/ This content is distributed under the terms of the Creative Commons Attribution 4.0 International license.

### Combining pSILAC with TMT labeling enables time-resolved quantitative proteome analysis during infection.

**FIG 1:**
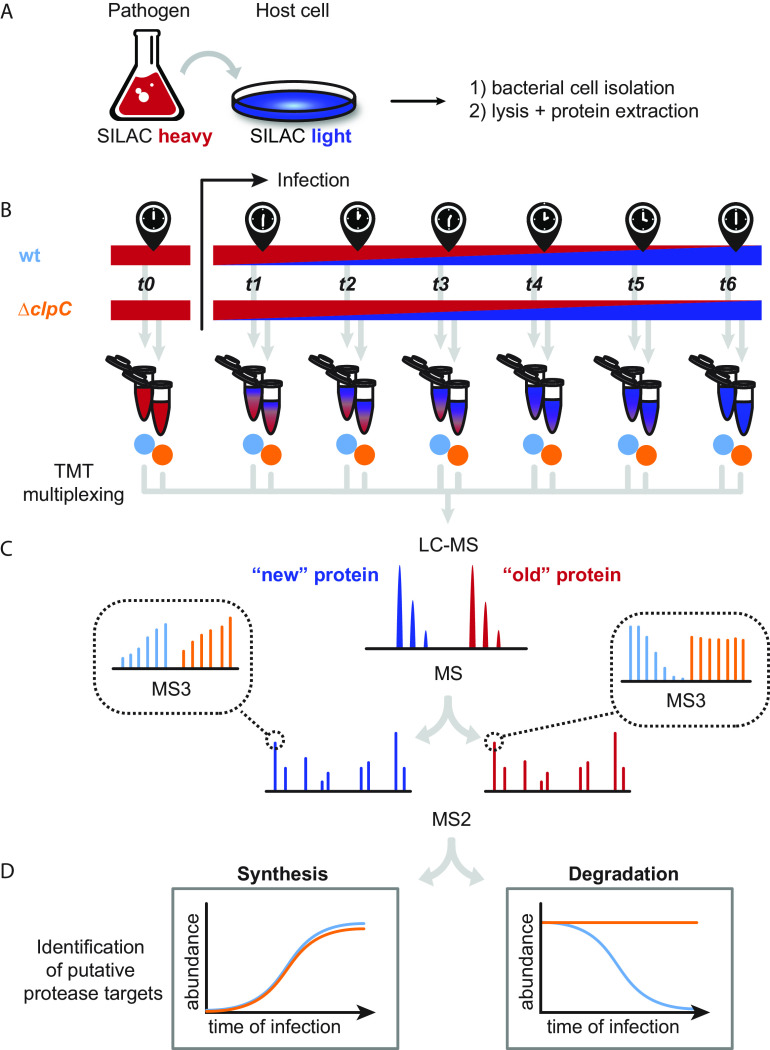
Overview of the experimental approach combining SILAC and TMT to study the temporal progression of bacterial adaptation to the host and to identify novel, ClpC-mediated proteolytic events in L. monocytogenes. (A) Lysine auxotrophic pathogenic bacterial strains are grown in SILAC heavy medium (red). A label switch to SILAC light medium (blue) is introduced upon Caco-2 cell infection with L. monocytogenes. (B) At different time points before and after infection (*t*_0_ to *t*_6_), bacteria are retrieved, and their cellular proteins are digested into peptides. TMT multiplexing allows analysis of all samples within one LC-MS run. wt (Δ*lysA*), light blue; Δ*clpC* (Δ*lysA*Δ*clpC*), orange. (C) Peptides from preexisting (heavy-labeled, in red) and newly synthesized proteins (light-labeled, in blue) are sequenced by MS2. TMT reporter ions are demultiplexed upon MS3 analysis, providing quantitative information for each time point and strain. wt (Δ*lysA*), light blue; Δ*clpC* (Δ*lysA*Δ*clpC*), orange. (D) Synthesis dynamics allow the identification of infection-specific protein translation. Stabilization of old proteins in the Δ*clpC* mutant enables the identification of putative novel ClpC substrates.

To discriminate between elevated expression levels and impaired proteolysis, we employed our pSILAC approach during an infection experiment. For in-depth quantitative proteome analysis of L. monocytogenes during infection, we infected Caco-2 cells with prelabeled L. monocytogenes grown in medium containing heavy lysine. The label switch started simultaneously with the infection process because the Caco-2 cells were cultured in standard medium containing solely the natural “light” amino acid variants. We analyzed samples from 7 time points to monitor protein synthesis and decay with high temporal resolution. The first sample was collected prior to infection, followed by 4 samples collected at 30-minute intervals to cover the internalization phase (time point 1 at 0.5 hours post-infection [hpi]), including cells attached to the host, and the early stages of infection at 1, 1.5, and 2 hpi. Two additional samples were collected at later time points (4 and 6 hpi). Combination of SILAC with TMT peptide labeling enabled analysis of all 14 samples (7 time points from wt and Δ*clpC* strain) in parallel within a single liquid chromatography-mass spectrometry (LC-MS) measurement (Fig. 1). In total, we detected 45,635 unique L. monocytogenes peptides in three biological replicates. Of those, 29,785 peptides contained at least one lysine and were therefore used for quantification. They correspond to 1,402 ± 10 unique heavy-labeled proteins and 1,061 ± 92 unique light-labeled proteins that were quantified in three biological replicates across all time points. In total, 1,692 unique proteins were detected, which covers 59% of the L. monocytogenes proteome. A total of 333 proteins were exclusively detected in their heavy-labeled form originating from synthesis before infection, while 53 proteins were detected only after infection. All quantitative data are available in Data Set S1 in the supplemental material.

10.1128/mSystems.00215-21.10 DATA SET S1 Quantitative proteomics data and the results from 1D annotation enrichment and Fisher’s exact test. Download Data Set S1, XLSX file, 7.2 MB. Copyright © 2021 Birk et al. 2021 Birk et al. https://creativecommons.org/licenses/by/4.0/ This content is distributed under the terms of the Creative Commons Attribution 4.0 International license.

Our in-depth quantitative proteome analysis showed very high reproducibility, with a mean Pearson correlation of 0.96 and 0.95 between biological replicates (Fig. S2A). For an overall assessment of the proteomic similarities and differences, we employed multidimensional scaling analysis (MDS), which confirmed the high similarity of biological replicates and revealed a clear separation of samples taken before and after infection (first dimension). The infection progression introduces variability in the proteome that is depicted in separation by time along the second dimension (Fig. S2B).

10.1128/mSystems.00215-21.2 FIG S2 (A) Scatter plot matrix comparing the correlation between biological replicates (Pearson’s correlation), visualized as log_2_ transformed protein abundances that were derived from the sum of the peptide reporter ion intensities. The diagonally oriented histograms illustrate the distribution of log_2_-transformed abundances across replicates. Displayed below the diagonal are the scatter plots comparing two replicates each. The red line in the graph represents a kernel density function fit. Pearson’s *r* values are shown for each comparison in the top triangle. (B) Multidimensional scaling plot after normalization. Download FIG S2, PDF file, 0.9 MB. Copyright © 2021 Birk et al. 2021 Birk et al. https://creativecommons.org/licenses/by/4.0/ This content is distributed under the terms of the Creative Commons Attribution 4.0 International license.

### Proteome adaptation of L. monocytogenes to the host environment.

**FIG 2:**
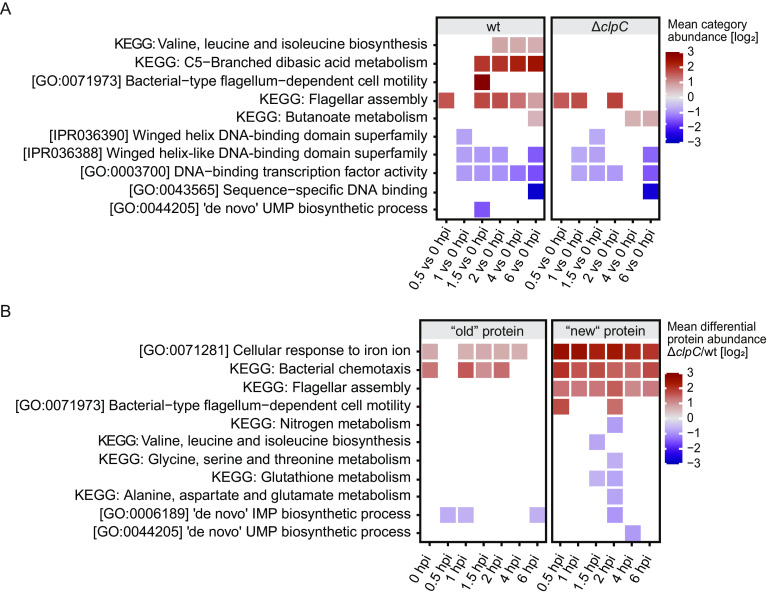
Pathways and categories demonstrating a significant abundance shift (>50% up- or downregulated [Δ*clpC*/wt], FDR < 0.1) upon infection compared to abundance at time point 0. Color coding represents the mean abundance shift indicating an increase in abundances over time in red and a decrease in abundances in purple. Only pathways and categories with a statistically significant shift are depicted in color, while blank spaces indicate the absence of a significant difference for that time point and category. The underlying data and the complete list of terms can be found in the Data Set 1 in the supplemental material. *n* = 3 independent biological experiments. wt (Δ*lysA*), Δ*clpC* (Δ*lysA*Δ*clpC*). (A) 1D annotation enrichment. Enrichment analysis of total protein abundances (cumulative heavy and light). Displayed are categories and pathways exhibiting >50% increase or decrease upon infection in the wt (left) or the Δ*clpC* mutant (right). (B) 1D annotation enrichment. Pathways and categories whose corresponding heavy-labeled proteins (old protein) or light-labeled proteins (new protein) display a significant change in abundance in the Δ*clpC* mutant compared to that in the wt strain.

To obtain an overview of the global proteome dynamics, we first aggregated for each protein heavy (“old”) and light (“new”) protein intensities. Based on these cumulative protein abundances, we found that in the wt strain, 938 out of the 1,692 identified proteins, i.e., 55%, changed in abundance during infection (one-way analysis of variance [ANOVA], false-discovery rate [FDR] ≤ 0.05). This indicates significant remodeling of the L. monocytogenes wt proteome in response to the host environment. A significant decrease of abundance compared to that at time point 0 was observed for proteins in categories related to DNA binding activity (terms: IPR036390, IPR036388, GO:0003700, GO:0043565) and pyrimidine biosynthesis (term: GO:0044205). Decreased pyrimidine biosynthesis upon infection was previously reported for intracellular L. monocytogenes (26). A significant increase of abundance was observed for proteins in pathways involved in motility (terms: KEGG: flagella assembly, GO:0071973) and branched-chain amino acid (BCAA) biosynthesis (term: KEGG: valine, leucine, and isoleucine biosynthesis) (Fig. 2).

Overall, 29 proteins were detected exclusively among the proteins newly synthesized upon infection in at least two biological replicates but not *in vitro* (time point 0), suggesting an infection-specific role. Among those are transporter proteins as well as proteins involved in motility (Table 1). Although not promoting motility *in vivo*, flagellar proteins are essential for adhesion and entry into nonphagocytic epithelial cells, such as Caco-2. Hence, their upregulation in the early stages of infection is consistent with previous research (27, 28). L. monocytogenes lacks the genes for nitrate reduction (29) and is therefore dependent on reduced nitrogen sources (e.g., amino acids or ammonia). Surplus ammonia is lethal to mammalian cells, so the amount of ammonia within the host cell cytosol is low (30, 31). Therefore, exclusive detection of proteins involved in the glutamine-glutamate synthesis pathway (GltAB; [Lmo1733 and Lmo1734]) among those proteins only present during infection could be expected.

**TABLE 1:** L. monocytogenes proteins exclusively detected *de novo* synthesized upon infection

Protein name and type	KEGG ID	UniProt ID	Protein function
Transporters			
PstB1	*lmo2495*	P63363	Phosphate import ATP-binding protein PstB 1
Lmo2760	*lmo2760*	Q8Y3S2	ABC transporter ATP-binding protein
Lmo2498	*lmo2498*	Q8Y4E8	Phosphate transport system permease protein
Lmo2346	*lmo2346*	Q8Y4T6	Amino acid ABC transporter ATP-binding protein
MntH	*lmo1424*	Q8Y773	Divalent metal cation transporter MntH
Hpt/UhpT	*lmo0838*	Q8Y8Q8	Sugar phosphate antiporter
Lmo1363	*lmo1363*	Q8Y7C2	ABC-type antimicrobial peptide transport system/permease
Nitrogen metabolism			
GltAB	*lmo1734*	Q8Y6F3	Glutamate synthase large subunit
GltAB	*lmo1733*	Q8Y6F4	Glutamate synthase subunit beta
Virulence factors			
Lmo1800	*lipA*	Q8Y696	Protein-tyrosine phosphatase
Lmo1786	*inlC*	Q8Y6A8	Internalin C
Lmo0514	*lmo0514*	Q8Y9L3	Internalin
Motility			
FlaA	*lmo0690*	Q02551	Flagellin
FliI	*lmo0716*	Q8Y926	H(+)-transporting two-sector ATPase
FliF	*lmo0713*	Q8Y929	Flagellar MS-ring protein FliF
FliD	*lmo0707*	Q8Y934	Flagellar hook-associated protein 2
FliP	*lmo0676*	Q8Y958	Flagellar biosynthetic protein FliP
DsbG	*lmo1059*	Q8Y859	DSB proteins
Other			
DsbA	*lmo0964*	Q8Y8E0	DSB proteins
Lmo0121	*lmo0121*	Q8YAK3	Phage tail protein
SpxA1	*lmo2191*	Q9RGX0	Regulatory protein Spx
Lmo2476	*lmo2476*	Q8Y4G7	Aldose 1-epimerase
Lmo1708	*lmo1708*	Q8Y6H6	Aminoglycoside N (3)-acetyltransferase
Lmo1998	*lmo1998*	Q8Y5Q8	Fructosamine deglycase
Lmo1597	*lmo1597*	Q8Y6T5	Unknown
Lmo1752	*lmo1752*	Q8Y6D5	Unknown
Lmo0119	*lmo0119*	Q7AP92	Unknown
LmaB	*lmo0117*	Q7AP94	Unknown
Lmo2112	*lmo2112*	Q8Y5F2	Unknown

The Δ*clpC* mutant underwent proteome remodeling upon infection similar to that of the wt. However, the Δ*clpC* mutant did not display an increased abundance of proteins involved in BCAA biosynthesis (Fig. 2A). Due to the importance of BCAA for virulence development (32, 33) and initiation of L. monocytogenes adaptation mechanisms (34, 35), this inability might be one explanation for the impaired virulence of the Δ*clpC* mutant.

Next, to gain a deeper understanding of the host adaption processes, we separately analyzed the data sets of preexisting (heavy) and newly synthesized (light) proteins. We determined the differential abundance between the Δ*clpC* mutant and wt (Δ*clpC*/wt) for each protein at each time point and conducted a pathway analysis. Proteins involved in bacterial chemotaxis (terms: KEGG: bacterial chemotaxis, KEGG: flagellar assembly) and the cellular response to iron (term: GO:0071281) exhibited higher abundances in the Δ*clpC* mutant in both old and new data sets (Fig. 2B). On the contrary, proteins involved in purine biosynthesis (term: GO:0006189) were less abundant in the Δ*clpC* mutant in both old and new data sets (Fig. 2B). This supports our hypothesis that an impairment in the formation of purine intermediates such as ATP and GTP might cause the previously observed decreased resistance of the Δ*clpC* mutant to a variety of stresses (8). Consistent with this hypothesis, several studies have previously provided evidence for the importance of *de novo* purine biosynthesis for virulence development of pathogenic bacteria (36–39).

Lower abundances of new proteins involved in pyrimidine (term: GO0044205), nitrogen, and amino acid biosynthesis indicates decreased synthesis of proteins within those pathways in the Δ*clpC* mutant compared to that in the wt strain (Fig. 2B).

### MurA1 accumulates in the Δ*clpC* strain in later stages of infection.

**FIG 3:**
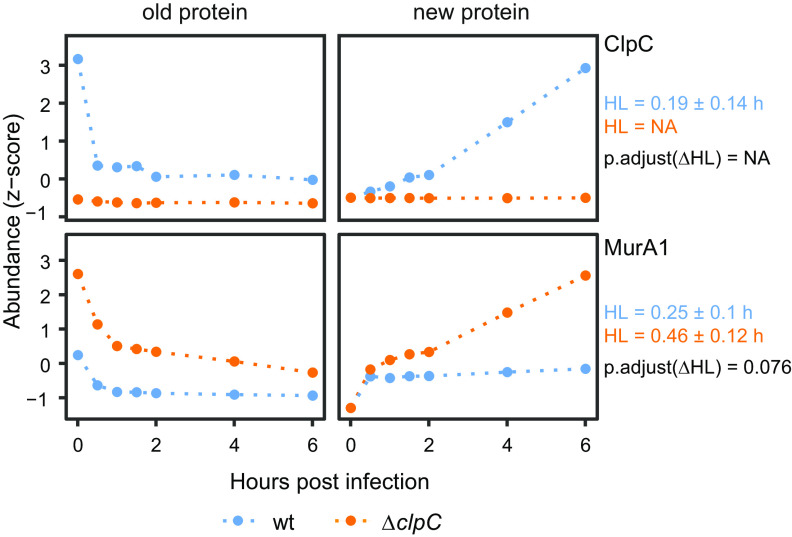
Degradation and synthesis dynamics of MurA1 and ClpC in the wt (Δ*lysA*, light blue) and the Δ*clpC* mutant (Δ*lysA*Δ*clpC*, orange). Plotted are hours post-infection against Z-scored protein abundances for degradation and synthesis dynamics. *n* = 3 independent biological experiments. *R*^2^ of curve fitting for half-life determination was >0.9.

We anticipated that direct ClpC substrates could be identified based on their increased abundance in the Δ*clpC* mutant compared to in the wt strain due to substrate accumulation caused by the lack of ClpCP-dependent proteolysis. To test this hypothesis, we analyzed the profiles of old and newly synthesized MurA1, a well-characterized ClpCP substrate in L. monocytogenes (40), which catalyzes the first committed step in peptidoglycan biosynthesis. The degradation profile of the old protein level revealed a general stabilization of MurA1 in the Δ*clpC* mutant throughout the experiment (Fig. 3). Furthermore, we observed that in the wt strain, newly synthesized ClpC increased at later stages of infection, whereas MurA1 levels remained constant. This is in stark contrast to the Δ*clpC* mutant, in which the absence of ClpC potentially prevents MurA1 from ClpCP-dependent degradation, which results in increased MurA1 abundance over time (Fig. 3). Identifying this negative correlation between MurA1 and ClpC abundance verifies the feasibility to identify ClpC targets during infection with our quantitative proteomics approach.

### Differential protein abundances pinpoint direct and indirect ClpC targets.

**FIG 4:**
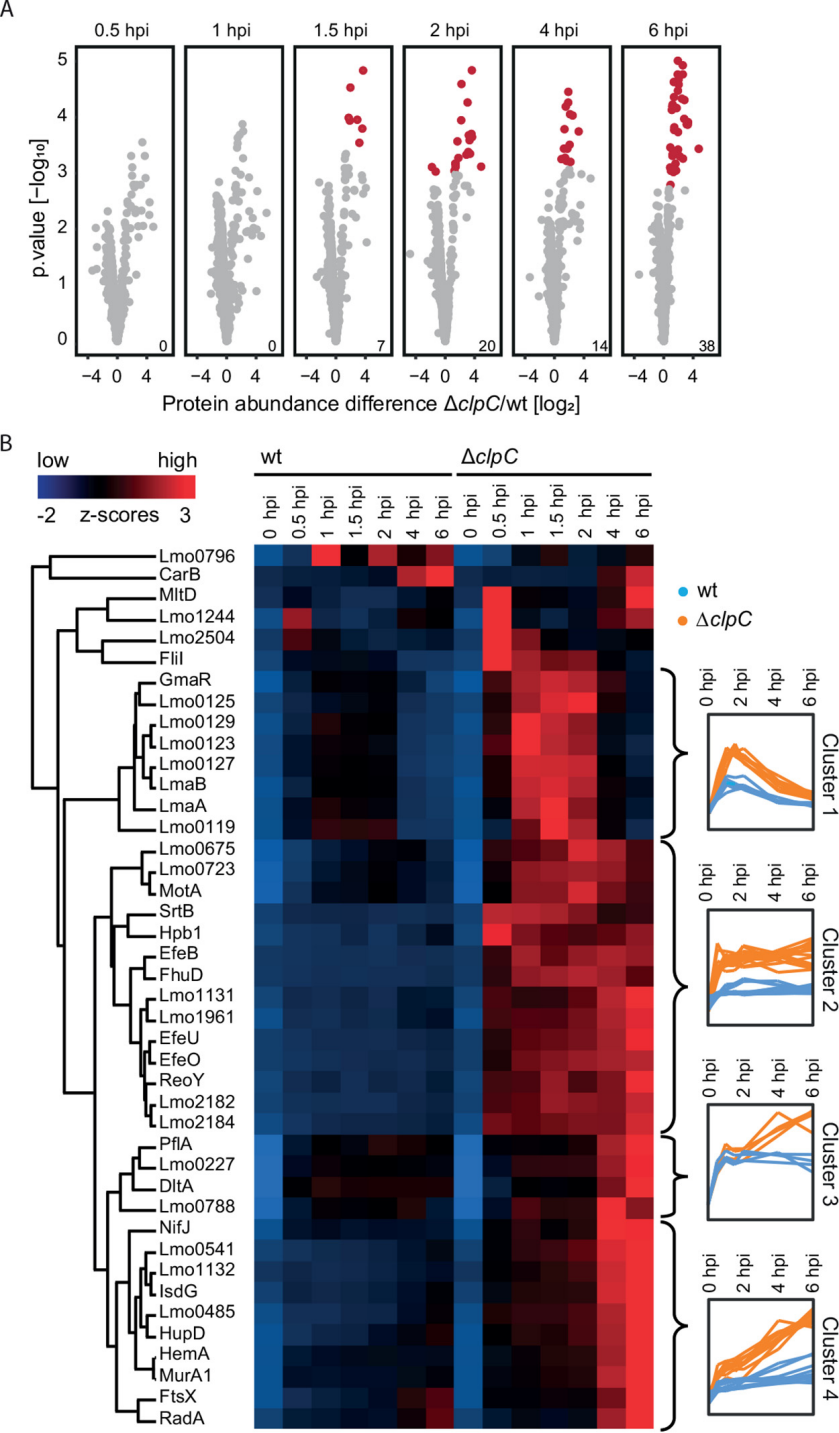
(A) Volcano plots showing abundance ratios of new proteins between the Δ*clpC* mutant (Δ*lysA*Δ*clpC*) and the wt strain (Δ*lysA*) and the statistical significance level. *n* = 3 independent biological experiments. Red dots indicate statistical significance (*P*_adj_ ≤ 0.05). Numbers in the bottom right corner indicate the number of proteins significantly different between the two strains at this time point. (B) (left) Heat-map visualizing hierarchical clustering based on Euclidean distance of Z-scored abundances of *de novo* synthesized proteins upon infection. (right) Profile plots displaying Z-scored protein levels for the corresponding clusters.

Next, we extended our search for putative direct and indirect ClpC targets by filtering for proteins that exhibited a statistically significant abundance difference between the two strains at any time point, which resulted in 109 old (Table S2) and 42 new proteins (Table S3) differentially abundant in the Δ*clpC* mutant compared to in the wt strain. Among the new proteins, we observed a significantly different abundance from 90 min onward (Fig. 4A). Unsupervised hierarchical clustering revealed four clusters of distinct profiles, indicating time point-specific changes in protein abundance (Fig. 4B). Note that the initial increase in abundance in all clusters resulted from the fact that no light-labeled proteins are present pre-infection.

10.1128/mSystems.00215-21.5 TABLE S2 *De novo* synthesized proteins with significant difference in abundance between the Δ*clpC* mutant and the wt. Values represent the log_2_ fold changes of abundances of a protein in the Δ*clpC* mutant compared to those in the wt strain. Values above 0 indicate an increase in protein abundance in the Δ*clpC* mutant compared to than in the wt. Download Table S2, PDF file, 0.6 MB. Copyright © 2021 Birk et al. 2021 Birk et al. https://creativecommons.org/licenses/by/4.0/ This content is distributed under the terms of the Creative Commons Attribution 4.0 International license.

10.1128/mSystems.00215-21.6 TABLE S3 Proteins with significant abundance difference in the “old” (heavy) proteins between the Δ*clpC* mutant and the wt strain. Values represent the log_2_ fold changes of abundances of a protein in the *clpC* deletion mutant compared to those in the wt strain. Download Table S3, PDF file, 0.5 MB. Copyright © 2021 Birk et al. 2021 Birk et al. https://creativecommons.org/licenses/by/4.0/ This content is distributed under the terms of the Creative Commons Attribution 4.0 International license.

Newly synthesized proteins in cluster 1 substantially increased in the early stages of infection (until 2 hpi), followed by a steady decrease from 2 to 6 hpi. This accumulation pattern indicates that these proteins perform a crucial cellular function during the early stages of infection but might not be required later. In the Δ*clpC* mutant, proteins in this cluster followed the same pattern; however, their abundances exceeded those detected in the wt strain, particularly at 2 hpi. One possible explanation is that the *clpC* deletion mutant is still able to initiate expression of those proteins to the same extent as the wt, but these proteins accumulated to higher levels due to impaired ClpCP-mediated proteolysis during the early stages of infection (until 2 hpi). Other proteases might compensate for this during later stages of infection. Alternatively, the expression levels of these proteins are higher in the Δ*clpC* mutant than in the wt strain, resulting in the observed enhanced accumulation in the Δ*clpC* mutant. The fact that 7/8 proteins of this cluster belong to the *lmaD*→*lmo0129* operon, which comprises 15 genes, indicates an indirect transcriptional effect of ClpC. Interestingly, we also found the regulator GmaR in this cluster, which controls the temperature-dependent transcription of flagellar motility genes by inhibiting the transcriptional repressor MogR (41). During temperatures above 37°C, MogR is released from GmaR, which in turn causes repression of flagellar motility genes and degradation of GmaR (42). Thus, ClpC influences GmaR abundance during infection, either directly through degradation, which is supported by existing evidence that GmaR is targeted for degradation by a protease (28), or indirectly on a transcriptional level. The increased GmaR level could explain the increased abundances of MogR-regulated gene products in the Δ*clpC* mutant that we have observed in the pathway analysis (Fig. 2A and B).

Proteins in clusters 2, 3, and 4 exhibited relatively constant protein abundance at a low level throughout infection in the wt strain, while their abundance in the Δ*clpC* mutant is elevated. For proteins in cluster 2, we observed an abundance consistently higher in the Δ*clpC* mutant compared to that in the wt strain across all time points. This observation implies an elevated expression of those proteins in the Δ*clpC* mutant rather than prolonged stability, where one would expect a steady increase in abundance over time. Consistent with this hypothesis, several proteins in cluster 2 are part of the Fur (ferric uptake regulator) regulon (SrtB-operon [SrtB, Lmo2182, Lmo2184], Hpb1, EfeOUB, FhuD) (43–45) or involved in chemotaxis and motility (Lmo0675, Lmo0723, MotA), with their expression being influenced by the abundance of the anti-repressor GmaR. As mentioned above, GmaR levels are increased in the Δ*clpC* strain. Interestingly, we did not observe differential protein abundance of the global regulator Fur itself. This gives rise to the hypothesis that Fur activity (but not its abundance), which depends on the binding to intracellular Fe^2+^ (46), might be affected by the *clpC* deletion. Consistent with this idea is the observation that a *clpC* mutant displays a strong growth defect during iron starvation (8, 9), supporting the hypothesis that iron metabolism may be impaired in a *clpC* mutant.

Levels of proteins in cluster 3 were constant in the wt strain, while they accumulated in the later stages of infection in the Δ*clpC* mutant. This accumulation of proteins in later stages could be due to either impaired ClpCP-mediated degradation or elevated expression in this mutant at later stages of infection. In cluster 4, we observed a slight but continuous increase of protein levels throughout infection in the wt strain. A similar increase is observed in the Δ*clpC* mutant, albeit with a steeper slope, which could also be caused by dependence on ClpCP-mediated degradation or an elevated expression at later stages of infection. Fifteen of these 42 new proteins differentially abundant between strains (Table S3) were also more abundant in the Δ*clpC* mutant *in vitro* (Fig. S1F and Table S1). The higher abundance of these proteins in the Δ*clpC* mutant is therefore not specific to infection. However, the inability to regulate their time point-specific accumulation in a wt manner could prevent the Δ*clpC* mutant from adapting to its host in the same way as the wt.

### Presence of ClpC influences global protein stability.

ClpC is one component of the ClpCP protease, and although ClpP, the other component, can associate and function with other Hsp100/Clp proteins (ClpX and ClpE in L. monocytogenes), we expect that putative ClpCP targets, whose proteolysis is solely dependent on the presence of ClpC, will accumulate and therefore exhibit a longer half-life in the Δ*clpC* mutant than in the wt. However, proteins targeted for ClpP-dependent degradation by another Hsp100/Clp protein in addition to ClpC are unlikely to be significantly stabilized in a Δ*clpC* mutant due to the remaining activity of ClpP with the other Hsp100/Clp protein (47, 48). Hence, we next focused our attention on the half-lives of heavy-labeled (old) proteins in order to identify additional potential ClpCP targets.

**FIG 5:**
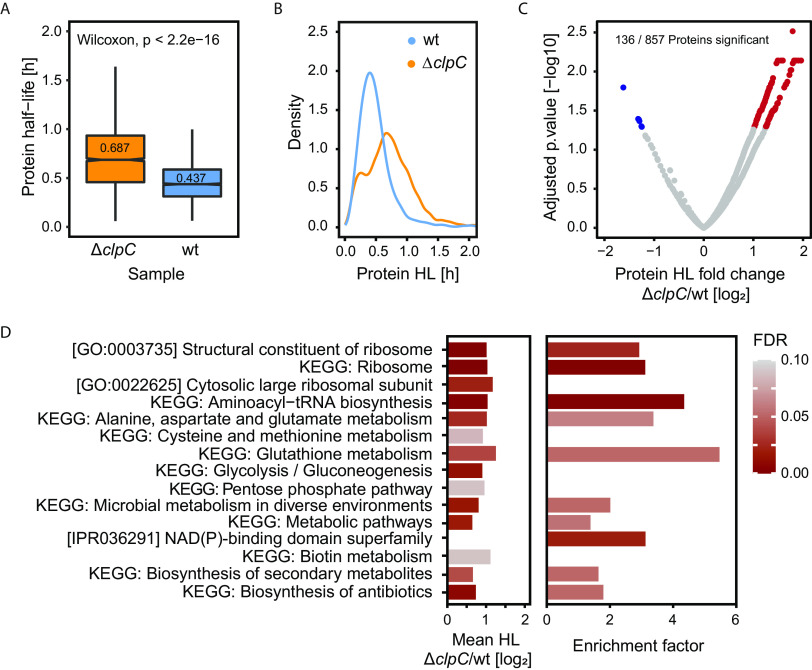
(A) Boxplot showing the distribution of half-lives in the wt strain (Δ*lysA*) and the Δ*clpC* mutant (Δ*lysA*Δ*clpC*) during infection. Wilcoxon rank-sum test: *P* < 2.2e−16. The *y* axis shows the half-lives in hours. *n* = 3 independent biological experiments. (B) Density plot reveals a bimodal distribution of half-lives in the Δ*clpC* strain. The *x* axis shows the half-lives in hours and the *y* axis shows the smooth density estimate (bandwidth = 0.05). *n* = 3 independent biological experiments. (C) Volcano plot showing delta protein half-lives and the statistical significance level (>2-fold difference in half-lives, *P*_adj_ ≤ 0.05). Red dots indicate proteins with significantly longer half-lives in the Δ*clpC* mutant compared to the wt strain. Blue dots indicate proteins with significantly shorter half-lives in the Δ*clpC* mutant compared to the wt strain. (D) Left bar plot: 1D annotation enrichment. Pathways with a significant shift in the mean delta HL of the corresponding proteins. Right bar plot: Results of Fisher’s exact test of the proteins with significantly longer half-lives in the Δ*clpC* mutant compared to the wt strain. The enrichment factor indicates to which extent proteins with significantly different half-lives in the two strains are enriched in the corresponding pathway. FDR   <   0.1. *n* = 3 independent biological experiments. The underlying data and the complete list of terms can be found in Data Set S1.

As expected, we observed a significant (Wilcoxon rank-sum test: *P* < 2.2e−16) shift in the mean protein half-life (wt: 0.44 h; Δ*clpC* mutant: 0.69 h), indicating prolonged protein half-lives in the Δ*clpC* mutant (Fig. 5A). The underlying bimodal distribution in the Δ*clpC* mutant indicates that the deletion only affects a subset of proteins within the cell and is not a general response caused by the *clpC* deletion itself (Fig. 5B). In total, we identified 131 proteins with significantly longer and 5 proteins with significantly shorter half-lives (Table S4) in the Δ*clpC* mutant when compared to the wt strain (FDR ≤ 0.05; cutoff log_2_ fold change of ≥1 or ≤−1) (Fig. 5C). Interestingly, only nine of those proteins also exhibited a statistically different abundance between the strains during infection, highlighting that the two analytical approaches we applied generated highly complementary data. While differences in half-lives between strains suggests constitutive degradation by ClpCP during infection in the wt strain, a difference in abundance would indicate a time point-specific alteration of protein abundance.

10.1128/mSystems.00215-21.7 TABLE S4 Proteins with significantly different half-lives in the Δ*clpC* mutant versus the wt. Fold change between half-lives indicates difference in half-lives between the Δ*clpC* mutant and the wt. Download Table S4, PDF file, 0.3 MB. Copyright © 2021 Birk et al. 2021 Birk et al. https://creativecommons.org/licenses/by/4.0/ This content is distributed under the terms of the Creative Commons Attribution 4.0 International license.

Unbiased pathway analysis based on the mean difference of protein half-lives between the two strains revealed that in the Δ*clpC* mutant, proteins in pathways associated with translation (terms: GO:0003735, GO:0022625, KEGG: aminoacetyl-tRNA biosynthesis, KEGG: ribosome), as well as carbon utilization (terms: KEGG: pentose phosphate pathway [PPP], KEGG: glycolysis/gluconeogenesis) and amino acid metabolism (Fig. 5D), have a significantly increased half-life. Similarly, enrichment analysis of the 131 proteins with a significantly longer half-life in the Δ*clpC* mutant revealed enrichment of the same pathways (i.e., translation, carbon utilization, and amino acid metabolism) (Fig. 5D).

Several of the proteins that display longer half-lives (PyrB, AroA, RpoB, GyrA, SecA2, Rnj, and GAPDH [Table S4]) have also been reported to exhibit longer half-lives in an isogenic *clpC* mutant in Bacillus subtilis under glucose starvation (48), supporting the idea that ClpCP is involved in their proteolysis in both B. subtilis and L. monocytogenes.

We did not observe a longer half-life of the *bona fide* ClpCP substrate MurA1, which is constantly degraded by ClpCP under optimal growth conditions and whose proteolysis is prevented by cell wall stress and remodeling (40, 49). Interestingly, previous research in L. monocytogenes has suggested cell wall remodeling during infection (50). Hence, this requirement of MurA1 activity for cell wall remodeling during these early stages of infection could be a possible explanation for the observation of almost no MurA1 degradation at this specific time point. Therefore, MurA1 would be stabilized in both strains, which could explain the similar half-lives of MurA1 that we have observed in the wt and Δ*clpC* strains during infection. MurA1 will be targeted again by ClpCP in the wt strain only when cell wall remodeling is completed at later time points, causing the observed accumulation of newly synthesized MurA1 in the Δ*clpC* mutant compared to that in the wt strain (Fig. 3), again emphasizing the importance of using complementary data analysis approaches to identify putative ClpCP targets.

### A potential role of ClpC in the downregulation of cell proliferation.

Based on the finding that proteins involved in pathways associated with translation exhibited significantly increased half-lives in the Δ*clpC* mutant, we hypothesized that ClpC is involved in adjusting the rate of cell proliferation to facilitate adaptation to the host environment. In line with this hypothesis, we found 9 aminoacyl-tRNA synthetases, 15 structural components of the ribosome, and several proteins involved in ribosome integrity or assembly to exhibit a longer half-life in the Δ*clpC* mutant (Table S4). Removal of proteins involved in translation is consistent with the finding that ribosomes synthesized during exponential growth are degraded under conditions in which growth decelerates to reduce translation and recycle released amino acids for enzyme synthesis (16, 18, 51, 52). Based on our data, we propose that ClpC is involved in this cellular process. Moreover, we observed stabilization of proteins involved in cell division (FtsA), peptidoglycan synthesis (MurD and MurE), replication (HolB), and genetic recombination (RecG, PriA, and RecN) in the Δ*clpC* mutant, supporting the hypothesis that ClpC is involved in the adjustment of the cell proliferation rate.

### ClpC is involved in the remodeling of carbon metabolism.

Our data further suggest a role for ClpC in remodeling carbon metabolism in response to the host environment. Intracellular L. monocytogenes utilizes glucose-6-phosphate (G6P), which is directed into the PPP, and glycerol, which feeds into lower glycolysis (53). Glucose-6-phosphate isomerase (Pgi) channels G6P into glycolysis by catalyzing the conversion of G6P to fructose-6-phosphate. A longer half-life of Pgi in the Δ*clpC* mutant suggests that ClpC might be involved in changing the carbon flux from glycolysis to the intracellular bipartite metabolism by targeting Pgi for degradation. Additionally, the intracellular bipartite metabolism does not require gluconeogenesis since the PPP fed by G6P is synthesizing essential precursors required for the biosynthesis of cell wall components and nucleotides. Consistent with the hypothesis that ClpC is involved in this metabolic shift, the putative pyruvate phosphate dikinase (Lmo1867), mediating the first gluconeogenic step from pyruvate, displayed a longer half-life in the Δ*clpC* mutant.

Furthermore, ClpC appears to be involved in the adaptation of the glycerol metabolism to its host environment. GlpK1, which is responsible for the first committed step in the glycerol metabolism (54), exhibited a longer half-life in the Δ*clpC* mutant. However, GlpK1 is not essential for survival in Caco-2 cells, where a second putative glycerol kinase (GlpK2) appears to take over some of its physiological function (54). We therefore speculate that GlpK1 is expressed in response to glycerol availability but is removed posttranslationally by ClpCP in response to an intracellular environment.

### ClpC is involved in the shedding of obsolete proteins.

Several proteins involved in metabolic pathways, which are not required during intracellular growth, displayed longer half-lives in a Δ*clpC* mutant, thereby suggesting that ClpC is involved in the downregulation of their cellular levels. Consistent with the observed ribosome degradation, these proteins no longer fulfill a required physiological function and are therefore recycled. This includes proteins needed for the acquisition of carbon sources not utilized during intracellular growth (e.g., malate [MaeA], PTS sugars [EI, HPrK/P]) and proteins involved in the *de novo* biosynthesis of amino acids that L. monocytogenes can scavenge from its host (53) (aromatic amino acids [AroA and AroE], aspartate-derived amino acids [e.g., AspB, putative aspartokinase Lmo1235, and aspartate-semialdehyde dehydrogenase Asd]).

### Interactive web-based application for visualization of the proteomics data.

To allow a more detailed analysis of the complete data, we developed a publicly available interactive web-based application that allows users to visualize the proteomics data (https://visualizations.mpusp.mpg.de/birk_01). Proteins can be selected based on their gene names, UniProt accession (55), KEGG ID no. (56), or protein function. Synthesis and decay dynamics for each protein quantified in this study can be visualized for the wt and the Δ*clpC* mutant.

## DISCUSSION

In this study, we combined pulsed SILAC during infection with TMT multiplexing to quantify the bacterial pool of old and newly synthesized proteins by quantitative mass spectrometry. Applying this methodology to intracellular bacteria provided unprecedented insight into host adaptation processes of L. monocytogenes on a proteome-wide scale and with high temporal resolution. We applied this novel approach to reveal putative targets of the protease complex ClpCP. This proof of principle illustrates how separate quantification of old and new proteins can illuminate mechanisms that would not be uncovered using conventional proteomic approaches.

The L. monocytogenes proteome during infection has previously been analyzed using 2D-DIGE proteomic analysis (26). However, this earlier study was based on total protein abundances and did not provide insights into synthesis and degradation. In 2013, a study using a SILAC-based approach in a Salmonella infection model led to a more accurate time-resolved analysis of adaptation mechanisms by assessing synthesis and degradation of proteins separately (21). A recent study demonstrated that the combination of pSILAC with isobaric TMT labeling facilitates comprehensive analysis of SARS-CoV-2-infected host cells over multiple time points (57). Our approach combines and expands on those substantial advances made in the field. We combined pulsed SILAC with TMT to investigate the proteome turnover during the temporal progression of bacterial adaptation to its host. In contrast to previous studies, our approach has the following two advantages. (i) Following proteome dynamics with high temporal resolution requires a more extensive sample series from multiple time points. In our case, we assessed the time-resolved adaptation of L. monocytogenes to its host by using 7 time points, starting from a very early stage of infection until 6 hpi. Previous studies largely neglected the temporal dynamics and focused on binary comparisons of samples collected before and after the infection, or were based on an experimental design, which did not allow analysis of protein decay. (ii) The reproducible and comprehensive analysis of this large data set was facilitated by using the multiplexing capabilities of TMT labeling. So far, the combination of SILAC and TMT has only been used to analyze protein turnover in eukaryotic cell culture systems, which are not limited regarding sample availability. Here, we demonstrate the feasibility to use this approach on intracellular bacterial cells.

Using this approach, we were able to investigate the role of a protease component during infection, providing insight into the role of ClpC in facilitating bacterial adaptation to the host. We found an accumulation of proteins involved in bacterial chemotaxis and the cellular response to iron and a decreased abundance of proteins involved in purine biosynthesis. We also found that the *clpC* deletion causes stabilization of proteins involved in cell proliferation and processes such as translation, carbon utilization, and amino acid metabolism. The identified putative substrates suggest that ClpC targets key players of cell proliferation to mediate adaptation to a new environment as the need for these proteins decreases under conditions of reduced growth. Impaired ability to downregulate the abundance of those proteins might severely affect the ability of the Δ*clpC* mutant to adapt to the host environment adequately.

Collectively, our results demonstrate the various direct and indirect roles that ClpC might fulfill in the metabolism, virulence development, and proteome remodeling of L. monocytogenes. Our data set complements earlier attempts to study metabolic changes of L. monocytogenes in response to the host and contributes to a better understanding of the role of protein degradation by ClpCP as a regulatory mechanism in bacterial virulence. This novel approach to studying the impact of a protease on the temporal remodeling of the proteome during infection can easily be adapted to investigate proteome remodeling under a variety of other conditions, including but not limited to different mutants or other pathogenic bacteria. We propose that this method can be instrumental to unraveling the effect of genetic perturbations or various environmental conditions (e.g., different host cell lines or drug treatments) on proteome remodeling in bacterial species.

## MATERIALS AND METHODS

### Bacterial culture.

Listeria monocytogenes EGD-e (serotype 1/2a) and isogenic gene deletion mutants were grown at 37°C with agitation at 180 rpm in listeria synthetic medium (LSM) (58) or brain heart infusion (BHI) medium (BD Bacto). Bacterial cell growth was monitored at regular time intervals by measuring the optical density of culture aliquots at 600 nm using a microplate reader (Synergy HTX multi-mode microplate reader, BioTek). The medium was inoculated from an exponential overnight culture to an optical density at 600 nm (OD_600_) of 0.05. Bacterial viability was monitored by determining CFU per milliliter (CFU/ml) over time on BHI agar plates (1.5% agar [wt/vol]). Erythromycin (Sigma-Aldrich) was added to the medium at a concentration of 5 μg/ml.

### Mutant construction.

To enable SILAC labeling of L. monocytogenes, we created a lysine auxotroph strain, which is dependent on external lysine for protein synthesis. We interrupted lysine production by deleting the gene for the enzyme diaminopimelate decarboxylase LysA (*lmo1952*/EC 4.1.1.20) that catalyzes the last step of lysine biosynthesis in L. monocytogenes.

Marker-less, in-frame deletions of *lysA* and *clpC* were constructed using the integrative shuttle vector pMAD (59). Up- and downstream regions (each approximately 800 bp) for the desired mutation site were amplified from chromosomal DNA of the parental strain (L. monocytogenes EGD-e) using primers OLEC8510 and OLEC8511 for the upstream region of *lysA*, primers OLEC7041 and OLEC7042 for the upstream region of *clpC*, primers OLEC8508 and OLEC8509 for the downstream region of *lysA*, and primers OLEC7043 and OLEC7044 for the downstream region of *clpC* (Table S5). The resulting fragments were cloned into pMAD using the isothermal (Gibson) assembly method (60) (Δ*lysA* deletion vector: pEC2415; Δ*clpC* deletion vector: pEC1974). The integrity of the plasmids was verified by PCR and DNA sequencing (Microsynth, Switzerland).

10.1128/mSystems.00215-21.8 TABLE S5 Primer list to generate the Δ*lysA* (wt) and Δ*lysA*Δ*clpC* (Δ*clpC*) mutant strains. The sequence annealing to the L. monocytogenes genome is indicated in italics and lowercase lettering. The sequence annealing to pMAD is indicated in uppercase lettering. Download Table S5, PDF file, 0.4 MB. Copyright © 2021 Birk et al. 2021 Birk et al. https://creativecommons.org/licenses/by/4.0/ This content is distributed under the terms of the Creative Commons Attribution 4.0 International license.

The Δ*lysA* deletion vector pEC2415 was introduced into the parental L. monocytogenes strain by electroporation as described before (61, 62). Erythromycin-resistant transformants were selected on solid BHI medium that contained erythromycin and 50 μg/ml X-Gal (Thermo Fisher Scientific). A single colony was grown in BHI shaking at 180 rpm at 30°C for several days to allow excision of the plasmid. Subsequently, several erythromycin-sensitive white colonies obtained from solid X-Gal-containing BHI medium were screened by PCR to identify mutants in which the second recombination step had resulted in the deletion of the *lysA* gene in the Δ*lysA* strain. The integrity of the strain was verified by PCR and DNA sequencing (Microsynth, Switzerland). The same procedure was performed for the Δ*clpC* deletion vector pEC1974, which was introduced into the Δ*lysA* mutant resulting in a double deletion mutant (Δ*lysA*Δ*clpC*). The integrity of both strains was also confirmed by whole-genome sequencing (Sequencing Core Facility, Max Planck Institute for Molecular Genetics, Germany). Throughout the article, the Δ*lysA* mutant is referred to as wt, while the Δ*lysA*Δ*clpC* double deletion mutant is referred to as Δ*clpC*. Table S6 lists all bacterial strains used in this study.

10.1128/mSystems.00215-21.9 TABLE S6 Strains used in this study. Download Table S6, PDF file, 0.5 MB. Copyright © 2021 Birk et al. 2021 Birk et al. https://creativecommons.org/licenses/by/4.0/ This content is distributed under the terms of the Creative Commons Attribution 4.0 International license.

### Eukaryotic growth conditions.

The Caco-2 cell line was obtained from the American Tissue Culture Collection (Caco-2/ATCC) and was used between passages 5 and 20. Caco-2 cells were maintained at 37°C and 5% CO_2_ in advanced Dulbecco modified minimum essential medium (DMEM; Gibco) supplemented with 10% (vol/vol) heat-inactivated fetal calf serum (FCS; Gibco), 1× GlutaMAX supplement (Gibco), 105 U/liter penicillin (Sigma-Aldrich), and 100 mg/liter streptomycin (Sigma-Aldrich). For infection experiments, Caco-2 cells were grown in the absence of antibiotics.

To investigate if the Δ*lysA* mutant (wt) is impaired in infecting Caco-2 cells, Caco-2 cells (passages 10 to 20) were seeded into 6-well plates and grown to 80% confluence. The Caco-2 cells were infected at a multiplicity of infection (MOI) of 20 to 50 in a final volume of 3 ml of medium with either the parental L. monocytogenes strain (EGD-e) or the *ΔlysA* mutant strain (wt). The inoculum was prepared from a culture in mid-logarithmic growth phase (OD_600_ = 0.4). After inoculation, the plates were centrifuged for 2 min at 600 × *g* at 37°C and maintained at 37°C and 5% CO_2_. After 4 h, the Caco-2 cells were washed with Dulbecco’s phosphate-buffered saline (DPBS; Thermo Fisher Scientific) and lysed using 0.1% (vol/vol) Triton X-100 in DPBS. Bacterial cells were plated in 1:10^5^ dilution on BHI agar plates, and CFU/ml were counted after approximately 20 h of incubation at 37°C.

To determine the effect of the *clpC* deletion on internalization, 50 μg/ml gentamicin was added to the cells infected with the Δ*lysA*Δ*clpC* (Δ*clpC*) and the Δ*lysA* (wt) strain at 30, 60, 90, and 120 min. For this step, Caco-2 cells were washed 3× with DPBS and the inoculum was replaced by medium containing 50 μg/ml gentamicin (Sigma-Aldrich) to kill extracellular bacterial cells. After 15 min, the cells were washed with DPBS and harvested using 0.1% (vol/vol) Triton X-100 in DPBS. Cells were plated in 1:10^5^ dilution on BHI agar plates, and CFU/ml were counted after approximately 20 h of incubation at 37°C.

### *In vitro* proteome comparison.

Cultures of the Δ*lysA*Δ*clpC* (named Δ*clpC*) and the Δ*lysA* (named wt) strains were harvested at mid-logarithmic phase and pelleted at 8,000 × *g* for 5 min at 4°C. The cell pellet was resuspended in 1 volume DPBS and pelleted again by centrifugation at 13,000 × *g* and 4°C for 1 min. The supernatant was removed and the cell pellet was resuspended in 150 μl lysis buffer (2% SDS [vol/vol] in 50 mM HEPES [pH 8], supplemented with 1× cOmplete EDTA-free protease inhibitor cocktail [Roche]). A total of 150 mg of BeadBeater glass beads (0.1 mm, Roth) was added to each sample and cells were lysed in a FastPrep-24 5G homogenizer (MP Biomedicals) by employing three homogenization cycles of 30 s each. Samples were then centrifuged for 10 min at 13,000 × *g* at room temperature. The supernatant was transferred to a fresh tube and the beads were washed with 200 μl lysis buffer. After an additional centrifugation step for 10 min at 13,000 × *g* at room temperature, the supernatant was transferred to the tube with the supernatant from the first step. Protein concentration was measured using the Micro BCA protein assay kit (Thermo Fisher Scientific) according to the manufacturer’s instructions. Samples were stored at −20°C prior to SP3 sample preparation.

### Pulsed SILAC for monitoring dynamic proteome remodeling.

Caco-2 cells (passages 9 to 12) were seeded into one-well plates (127.8 by 85.5 mm, Greiner) and cultivated at 37°C and 5% CO_2_ in growth medium consisting of advanced DMEM supplemented with 10% (vol/vol) heat-inactivated FCS and 1× GlutaMAX supplement. At 80% confluence, the cells were infected with the respective L. monocytogenes strains at an MOI of 10 in a final volume of 10 ml of medium. The inoculum was prepared from a culture in mid-logarithmic growth phase grown in LSM containing heavy lysine (Silantes, #211604102). Heavy lysine incorporation {∑PSM_[heavy K]_/[∑PSM_(heavy K)_ + ∑PSM_(light K)_]} was 99.7% (where PSM is peptide-spectrum-matches). After inoculation, the plates were centrifuged for 2 min at 600 × *g* at 37°C and maintained at 37°C and 5% CO_2_. The first sample was harvested after 30 min. At the same time, the inoculum containing free-floating bacterial cells was removed from the remaining cultures and exchanged for 10 ml medium containing 50 μg/ml gentamicin to kill extracellular bacterial cells. After another 15 min, the medium was exchanged for medium containing 10 μg/ml gentamicin. Samples from five additional time points were harvested at 1, 1.5, 2, 4, and 6 h postinfection. For harvesting, the cells were rinsed three times with 5 ml DPBS at room temperature. The Caco-2 cells were thereafter lysed by adding 5 ml 0.1% (vol/vol) Triton X-100 in DPBS. The organic material consisting of Caco-2 cell debris and intact bacterial cells was pelleted at 8,000 × *g* for 5 min at 4°C and resuspended in modified radioimmunoprecipitation assay (RIPA) buffer (25 mM Tris-HCl [pH 7.6], 150 mM NaCl, 1% Triton X-100 [vol/vol], 1% SDS [vol/vol]). Intracellular bacteria were purified using differential centrifugation. Briefly, the resuspended pellet was centrifuged at 600 × *g* for 10 min at 4°C to remove Caco-2 cell debris. The supernatant was transferred to a new tube and centrifuged at 13,000 × *g* for 1 min at 4°C to pellet bacterial cells. The supernatant was removed and the pellet was resuspended in 150 μl 50 mM HEPES (pH 8) containing 1× cOmplete EDTA-free protease inhibitor cocktail (Roche). A total of 150 mg of BeadBeater glass beads, 0.1 mm (Roth), were added per sample and cells were lysed using a FastPrep-24 5G homogenizer (MP Biomedicals) employing three homogenization cycles of 30 s each. Samples were incubated on ice for 5 min followed by a 10-min centrifugation step at 13,000 × *g* at 4°C. The supernatant containing the soluble protein fraction was transferred to a fresh tube and the beads were washed with 200 μl 50 mM HEPES (pH 8) containing 1× cOmplete EDTA-free protease inhibitor cocktail. The samples were centrifuged for 10 min at 13,000 × *g* and 4°C, and the supernatant was added to the soluble protein fraction. Protein concentration was measured using the Micro BCA protein assay kit (Thermo Fisher Scientific). Samples were stored at −80°C prior to SP3 sample preparation.

### SP3 sample preparation.

A modified SP3 protocol (63) was used for mass spectrometry sample preparation. A total of 15 μg protein from each sample was mixed with 4× lysis buffer (50 mM HEPES [pH 8; VWR], 4% SDS (vol/vol) (Applichem), 160 mM 2-chloroacetamide (CAA), 40 mM Bond-Breaker Tris(2-carboxyethyl)phosphine hydrochloride [TCEP] solution [Thermo Fisher Scientific]) to a final concentration of 1× absolute volume and incubated for 5 min at 95°C. Samples were cooled to room temperature and Benzonase nuclease (0.5 units per μg of protein, Merck) was added to each sample. Nucleic acids were degraded during incubation for 30 min at 37°C while shaking at 500 rpm. SP3 beads (1:1 mixture of hydrophobic and hydrophilic carboxyl-coated Sera-Mag SpeedBeads catalog no. 45152105050250 and 65152105050250, GE Healthcare) were added to the samples in a bead/protein ratio of 10:1 (wt/wt). Samples were mixed by pipetting, and anhydrous acetonitrile (Thermo Fisher Scientific) was added to a final concentration of 50% (vol/vol). To allow protein aggregation on the beads, the samples were incubated for 20 min at room temperature. Subsequently, the beads were collected on a magnetic stand for 5 min and the supernatants were discarded. The beads were washed 3 times with 80% (vol/vol) ethanol for 3 min. Beads were air-dried at room temperature for 10 min to allow evaporation of residual ethanol. The beads were thereafter resuspended in 30 μl digestion buffer (50 mM triethylammonium bicarbonate) containing trypsin (Serva) and LysC (Wako) in a 1:50 (wt/wt) enzyme-to-protein ratio. Protein digestion was carried out at 37°C for 14 h in a PCR cycler. The beads were collected on a magnetic stand for 5 min and the supernatants containing the peptides were collected. For internal reference scaling, equal amounts of peptides from all samples were pooled. The peptides were then dried using a vacuum concentrator.

### Tandem-mass-tag labeling.

For proteome comparison during infection, we used 16plex TMTpro (Thermo Fisher Scientific) for multiplexing. Fourteen channels were used for each biological replicate. The 15th channel was selected for the internal standard consisting of equal amounts of protein from all samples across strains and biological replicates. For *in vitro* proteome comparison, we used 6plex TMT (Thermo Fisher Scientific) for multiplexing.

We optimized the labeling conditions based on the recommendations of Zecha et al. (22). Briefly, the final concentrations of TMT reagents, peptides, and absolute labeling volume were tailored toward the minimum amounts of proteins available for this study. Identical amounts of peptides for each time point were adjusted to a final concentration of 1 μg/μl in 50 mM HEPES buffer (pH 8.5). TMT labeling reagents were dissolved in acetonitrile to a concentration of 59 mM. Final TMT concentration during labeling was 11.8 mM, and the final acetonitrile concentration was 20% (vol/vol). Labeling occurred for 1 h at room temperature. The labeling process was quenched by addition of hydroxylamine (5% in water) to a final concentration of 0.02% (vol/vol) followed by 5 min of incubation at room temperature. Samples for all TMT channels of one experiment were combined after the labeling, dried in a vacuum concentrator at room temperature, and fractionated by high pH reversed phase chromatography as described below.

### High pH peptide fractionation.

Pooled and dried samples were resuspended in 20 μl 2% (vol/vol) acetonitrile for fractionation. Basic reversed phase fractionation was performed on a quaternary Agilent 1290 Infinity II UPLC system equipped with a Kinetex Evo-C_18_ column (150 by 2.1 mm, 2.6 μm, 100 Å, Phenomenex) that was operated at 40°C. Solvent A consisted of high-performance liquid chromatography (HPLC)-grade H_2_O, solvent B consisted of 100% acetonitrile, and solvent C consisted of 25 mM ammonium bicarbonate. Fractionation was carried out at a constant flow rate of 100 μl/minute using a linear gradient from 2 to 25% (vol/vol) acetonitrile within 50 min, followed by column washing and equilibration. Over the whole gradient, solvent C was kept constant at 10% (vol/vol). In total, 32 fractions with 200 μl per fraction were collected in conical 96-well plates (Greiner). The organic solvent was removed in a vacuum concentrator (default settings for organic solvents) for 1 h and fractions were concatenated into 8 final samples as described before (64). Peptides were acidified with formic acid (Thermo Fisher Scientific) to a final concentration of 1% (vol/vol), desalted using OASIS HLB 96-well cartridges (Waters), dried down at room temperature, and resuspended in 30 μl 2% (vol/vol) acetonitrile, 0.1% (vol/vol) trifluoroacetic acid (TFA) prior to mass spectrometry (MS) analysis.

### Mass spectrometry.

Peptide fractions were analyzed on an Orbitrap Fusion Lumos (Thermo Fisher Scientific) that was coupled to a 3000 RSLC nano UPLC (Thermo Fisher Scientific). Samples were loaded on a pepmap trap cartridge (300 μm inside diameter [i.d.] by 5 mm, C_18_, Thermo Fisher Scientific) with 2% (vol/vol) acetonitrile, 0.1% (vol/vol) TFA at a flow rate of 20 μl/min. Peptides were separated over a 50 cm analytical column (Picofrit, 360 μm outside diameter [o.d.], 75 μm i.d., 10 μm tip opening, noncoated, New Objective) that was packed in-house with Poroshell 120 EC-C_18_, 2.7 μm (Agilent). Solvent A consists of 0.1% (vol/vol) formic acid in water. Elution was carried out at a constant flow rate of 250 nl/min using a 180-minute method: 8 to 33% (vol/vol) solvent B (0.1% [vol/vol] formic acid in 80% [vol/vol] acetonitrile) within 120 min, 33 to 48% (vol/vol) solvent B within 25 min, 48 to 98% (vol/vol) solvent B within 1 min, followed by column washing and equilibration. A spray voltage of 2.2 kV was applied via liquid junction. The Fusion Lumos mass spectrometer was equipped with a FAIMS Pro device, which was operated at standard resolution using three alternating compensation voltages (CVs) of −40 V, −60 V, and −80 V. The cycle time for each CV was set to 1 s. Data acquisition was carried out using an MS3-based data-dependent method. Advanced peak determination was deactivated. MS survey scans were acquired from 375 to 1,500 *m/z* in profile mode at a resolution of 120,000. The automatic gain control (AGC) target was set to 4e5 charges, allowing a maximum injection time of 50 ms. The RF lens was operated at 35%. Peptides with charge states 2 to 6 were subjected to collision-induced dissociation (CID) fragmentation (fixed collision energy [CE] = 35%, AGC = 1e4) and analyzed in the linear ion trap at a scan rate of 125,000 Da/second. The maximum injection time for MS2 scans was set to 50 ms. The isolation window for peptides with charge states 2, 3, and ≥4 was set to 1.2, 0.7, and 0.5 *m/z*, respectively. For MS3 acquisition, synchronous precursor selection (SPS) was enabled to select 10 fragment ions for higher-energy collisional dissociation (HCD) fragmentation (isolation window = 2 *m/z*, normalized collision energy [NCE] = 45%, AGC = 1e5, maximum injection time = 105 ms). MS3 scans were acquired in the Orbitrap from 100 to 500 *m/z* at a resolution of 50,000. Precursors were dynamically excluded for 45 s. MS analysis of TMT 6plex samples was carried out using the same method with the following exceptions for MS3 acquisition: HCD collision energy was set to 65%, Orbitrap scan resolution was set to 15,000 with a maximum injection time of 22 ms.

### MS raw data processing.

Raw files were processed with Proteome Discoverer 2.4 (Thermo Fisher Scientific). Briefly, peak lists were extracted from raw files and searched using SEQUEST HT against UniProt databases (55) containing protein sequences of Homo sapiens (taxonomy ID no. 9606, version 190111) and Listeria monocytogenes (taxonomy ID no. 169963, version 190219) and a database containing sequences of common contaminants (derived from MaxQuant v.1.6.0.1). Trypsin/P was set as enzyme specificity, allowing a maximum of two missed cleavages. The minimum peptide length was set to 7 amino acids. Carbamidomethylation (+57.021 Da) on cysteine was set as fixed modification, and oxidation (+15.995) of methionine was set as variable modification. In order to enable TMT quantification of SILAC-labeled proteins, the TMT modifications were set as follows: TMTpro (+304.207 Da) or TMT 11plex (+229.163 Da), respectively, were set as fixed modification on peptide N termini. Two variable modifications were defined to allow for TMT labels on light (+304.207 Da [TMTpro] or +229.163 Da [TMT 11plex]) and heavy (+310.227 Da [TMTpro] or +235.183 Da [TMT 11plex]) lysines, respectively. A maximum of four variable modifications was allowed per peptide. Percolator (65) was used to calculate *q* values for identifications from the target/decoy search. Mass tolerances for MS1 and MS2 were set to 10 ppm and 0.6 Da, respectively. TMT reporter ion abundances were quantified within a 20-ppm window and calculated based on the raw intensities of the most confident centroid. Quan value correction was applied to correct for reagent isotope impurities. The resulting table of peptide-spectrum-matches (PSMs) was exported as plain text file for downstream data analysis in R. The mass spectrometry proteomics data have been deposited to the ProteomeXchange Consortium via the PRIDE partner repository (66) with the data set identifier PXD023608.

### Data analysis and normalization.

Data filtering and normalization were carried out in R. Peptide-spectrum-matches (PSMs) were filtered to remove contaminants, peptides with inconsistent TMT labeling, peptides with inconsistent SILAC labeling state, peptides derived from the human host cells, and peptides with low-quality MS3 quantification, accepting a maximum of 25% MS1 isolation interference for quantification. Human cell contamination averaged 35 ± 8%, as determined from the TMT reporter ion intensities, and was independent of time point and strain type (Fig. S3). The synchronous precursor selection (SPS) identity threshold was set to 50%. PSMs corresponding to the same peptide sequence were aggregated, and peptides without a lysine were removed because they cannot be used to distinguish heavy (preexisting) and light (newly synthesized) proteins. The false-discovery rate (FDR) for peptide identification was set to 1% in all analyses, i.e., PSMs with a percolator *q* value of >9.99 × 10^−3^ were discarded. Sample loading (SL) normalization was applied to adjust the total intensity of each channel to the average total intensity across all channels. We applied internal reference scaling (IRS) (67) to correct for batch effects originating from combination of data from several TMT sets. The internal standard, consisting of a peptide mixture of each sample, was used to normalize reporter ion intensities of proteins between different TMT experiments. The summed reporter ion measurements of the internal reference for each protein in all experiments were averaged to calculate an individual reference value for each protein. Individual intensity-scale measurements are thereby preserved. We corrected for compositional bias in the data set using upper quartile (UQ) normalization (68) (edgeR package, https://bioconductor.org/packages/release/bioc/html/edgeR.html) (69). To test for reproducibility, the Pearson correlation of protein abundances between biological replicates was determined using the psych package (https://cran.r-project.org/web/packages/psych/index.html).

10.1128/mSystems.00215-21.3 FIG S3 Human contamination in percent for the three biological replicates of both strains at each time point postinfection. Average contamination is 35 ± 8%. Contamination is independent from time point and type of strain (wt [Δ*lysA* strain] and Δ*clpC* [Δ*lysA*Δ*clpC* mutant]). Download FIG S3, PDF file, 0.4 MB. Copyright © 2021 Birk et al. 2021 Birk et al. https://creativecommons.org/licenses/by/4.0/ This content is distributed under the terms of the Creative Commons Attribution 4.0 International license.

### Determination of protein half-lives.

For all proteins labeled with heavy lysine, representing proteins that existed before the label switch, we calculated the relative intensities. The protein intensity of each protein at each time point was divided by the intensity of the protein at time point 0. Thereby, the protein intensities at time point 0, resembling the starting point of the protein turnover, were set to 1. The data were filtered to exclude proteins that have a relative abundance of >1 in any of the time points after the label switch (time points 1 to 6). Additionally, we also removed those proteins with a positive linear regression slope. These additional filtering steps removed less-abundant proteins (approximately 6.7%) that were inconsistently quantified. For curve fitting, we used the R package minpack.lm (https://CRAN.r-project.org/package=minpack.lm) and implemented a model that was introduced by Welle et al. (23) and later adapted by Zecha et al. (22). Protein degradation was modeled using the following function: *y* = (*A*_degradation_ − *B*_degradation_) × *e*^(−*K*_degradation_ × *x*)^ + *B*_degradation_, with the starting values *A*_degradation_ = 1, *B*_degradation_ = 0.3, *K*_degradation_ = 0.01, the lower modeling constraints *A*_degradation_ = 0, *B*_degradation_ = −2, *K*_degradation_ = −1, and the upper modeling constraints *A*_degradation_ = 3, *B*_degradation_ = 2, *K*_degradation_ = 15. *y* is the fractional abundance of the heavy protein at each time point relative to time point 0. *e* refers to Euler’s number. *K*_degradation_ is the degradation rate constant. *A*_degradation_ refers to the maximum of the fitting curve and is 1 in an ideal case. *B*_degradation_ accounts for a potential fitting curve offset which ideally is zero. *x* represents the time in hours (22). To assess the goodness-of-fit (pseudo *R*^2^) for the fitted functions, we used the R package rcompanion (https://CRAN.r-project.org/package=rcompanion) and the Nagelkerke method to compare a nonlinear model to a null model. The minpack.lm package was used to fit a null model for the same time points as for the actual data: *y* = function(*x*, *j*){*j*} with the starting value *j* = 1. Fitting results were discarded if the fitting parameters did not meet the constraints 0.5 ≤ *A* ≤ 2, −1 ≤ *B* ≤ 1, *K* < 12. Proteins with a pseudo *R*^2^ of ≤0.9 were discarded to eliminate low-quality fits. Half-lives (HL) were calculated from the parameter *K* using the formula HL = log_2_/*K*. Of note, our statistical model does not take cell proliferation into account. The calculated protein half-lives therefore allow comparison of wt and *ΔclpC* strains but should not be interpreted as generalizable absolute values. Statistical significance of delta half-lives (HL_Δ_*_clpC_*/HL_wt_) was assessed using the limma package (https://bioconductor.org/packages/release/bioc/html/limma.html). Briefly, log_2_-transformed delta half-lives were tested for difference from zero by a moderated *t* test. Resulting *P* values were corrected for multiple testing by the method of Benjamini-Hochberg. Proteins with a >2-fold difference in half-lives and a *P*_adj_ of ≤0.05 were considered significant.

### Analysis of *de novo* protein synthesis.

For all proteins labeled with light lysine, representing proteins that were *de novo* synthesized after the infection induced label switch, we calculated the relative intensities. For each protein, we subtracted the background noise intensity at time point 0 from the intensities at the other time points. To determine the differences in *de novo* synthesis between the two strains, we calculated for each protein at each time point the ratio between the intensities (Δ*clpC* [Δ*lysA*Δ*clpC*]/wt [Δ*lysA*]). The ratios were then log_2_ transformed and tested for difference from zero using limma as described above.

### Statistical and pathway analysis.

KEGG pathways (56) were downloaded using the R package KEGGREST (https://bioconductor.org/packages/release/bioc/html/KEGGREST.html). Additional protein annotations were obtained from ListiWiki (70). Gene Ontology and Interpro data were retrieved from UniProt (55) using the R package UniProt.ws (https://bioconductor.org/packages/release/bioc/html/UniProt.ws.html).

Statistical analysis of functionally related proteins was conducted within Perseus 1.6.2.3 (71). A one-way ANOVA (fudge factor [*s*_0_] of 0.1 [72]; permutation-based FDR ≤ 0.05) was applied to test for significant changes of cumulative protein abundances. Categorical annotations of candidate proteins in either light or heavy data set (significant as determined by limma) were tested for statistical significance using Fisher’s exact test (correction for multiple testing by Benjamini-Hochberg). Unbiased analysis of categorical annotations was carried out using one-dimensional (1D) annotation enrichment (73).

### Web-based application for visualization of the proteomics data.

The interactive web-based application that visualizes the presented proteomics data supports all major web browsers. The app was implemented in Python (v3.6.9) using Flask (v1.1.2) and Dash (v1.16.1) (Plotly Technologies Inc., 2015) for the web framework. The Python package Pandas (v1.1.2) was used for data handling. The reverse proxy server Nginx (v1.16.1), which handles the requests, was connected to the web framework by Gunicorn (v20.0.4). UniProt (55) and KEGG (56) identifiers were mapped using the Python package bioservices (v1.7.9). The web-based application for visualization of the proteomics data is available at https://visualizations.mpusp.mpg.de/birk_01.

### Data availability.

The mass spectrometry proteomics data have been deposited to the ProteomeXchange Consortium via the PRIDE partner repository (66) with the dataset identifier PXD023608.
