# Supplementary figures and images for: Time-Resolved Proteome Analysis of Listeria monocytogenes during Infection Reveals the Role of the AAA+ Chaperone ClpC for Host Cell Adaptation
Source: mSystems. 2021 Aug 3;6(4):e00215-21. doi: 10.1128/mSystems.00215-21 (PMC8407217; doi:10.1128/mSystems.00215-21)

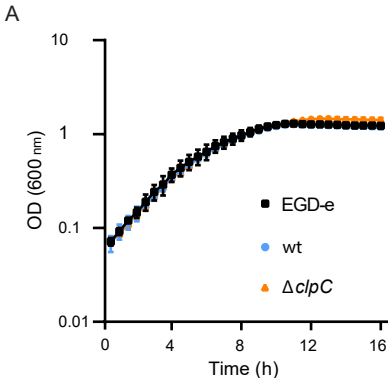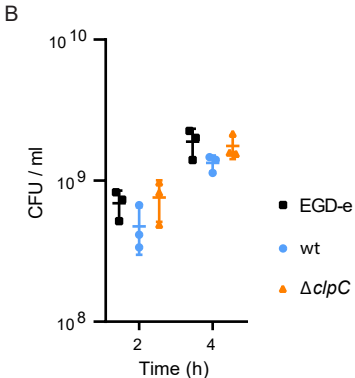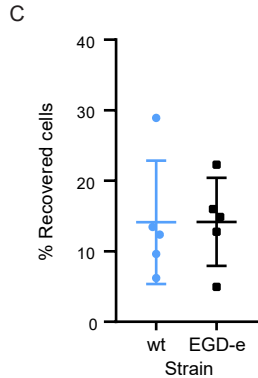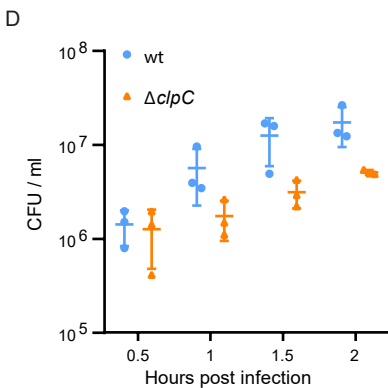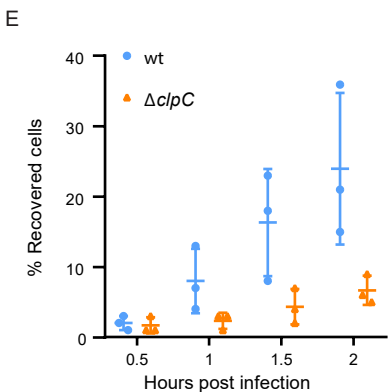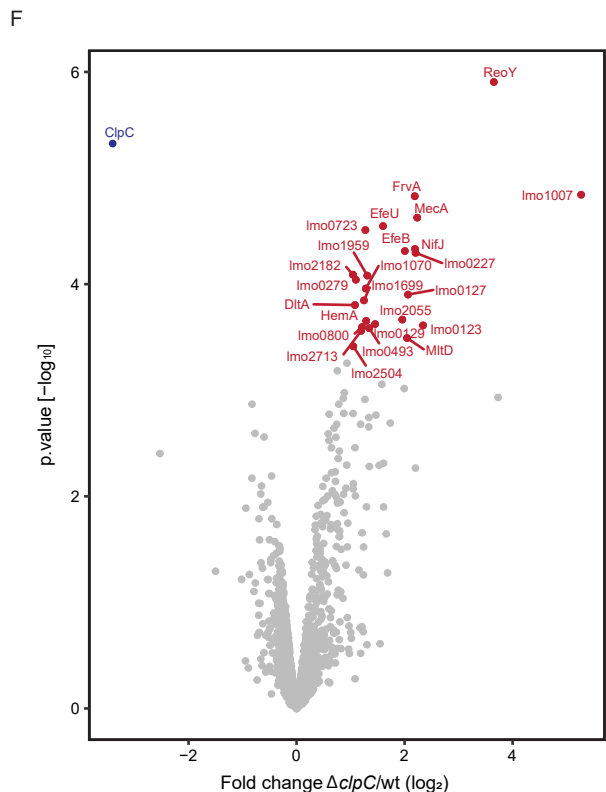

Supplement: FIG S1 [file msystems.00215-21-sf001.pdf]

A

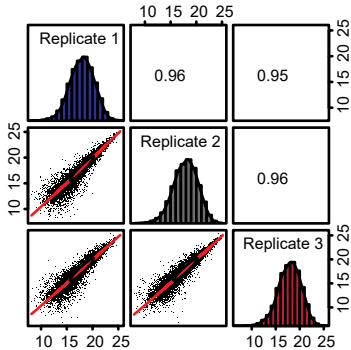

B

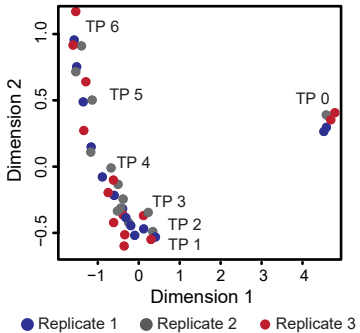

Supplement: FIG S2 [file msystems.00215-21-sf002.pdf]

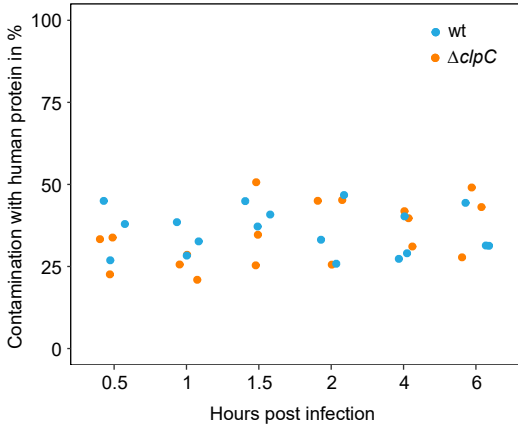

Supplement: FIG S3 [file msystems.00215-21-sf003.pdf]
