# Supplementary material for: Time-Resolved Proteome Analysis of Listeria monocytogenes during Infection Reveals the Role of the AAA+ Chaperone ClpC for Host Cell Adaptation
Source: mSystems. 2021 Aug 3;6(4):e00215-21. doi: 10.1128/mSystems.00215-21 (PMC8407217; doi:10.1128/mSystems.00215-21)
Supplement: TABLE S1 [file msystems.00215-21-st001.pdf]

Protein name	KEGG ID	Uniprot ID	Function
**Cell shape and surface determinants**			
DltA	lmo0974	Q8Y8D4	D-alanine--D-alanyl carrier protein ligase
MltD	lmo2522	Q8Y4C8	Murein transglycosylase D
**Chemotaxis**			
lmo0723	lmo0723	Q8Y919	Methyl-accepting chemotaxis protein
lmo1699	lmo1699	Q8Y6I5	Chemotaxis protein
lmo2504	lmo2504	Q8Y4E2	Involved in biofilm formation
**ClpC protease related**			
ReoY	lmo1921	Q8Y5Y2	Effector protein of MurA1 degradation by ClpCP
ClpC	lmo0232	Q8YAB6	Endopeptidase Clp ATP-binding chain C
MecA	lmo2190	Q9RGW9	Effector protein of ComK degradation by ClpCP in *B. subtilis*
**Cellular response to iron**			
EfeU	lmo0365	Q8YA02	High-affinity iron transporter
EfeB	lmo0367	Q8YA00	Deferrochelatase/peroxidase
lmo2182	lmo2182	Q8Y587	Heme transporter analogous to IsdDEF
lmo1959	lmo1959	Q8Y5U6	Ferrichrome-binding periplasmic protein precursor
HemA	lmo1557	Q8Y6X4	Glutamyl-tRNA reductase
FrvA	lmo0641	Q8Y992	Fur-regulated virulence protein
**Adaptation to low oxygen conditions**			
lmo0279	lmo0279	Q8YA80	Anaerobic ribonucleoside triphosphate reductase
NifJ	lmo0829	Q8Y8R6	Pyruvate-flavodoxin oxidoreductase
**Other**			
lmo0123	lmo0123	Q8YAK1	Unknown
lmo0127	lmo0127	Q8YAJ7	Unknown
lmo0129	lmo0129	Q8YAJ6	Unknown
lmo1007	lmo1007	Q92D15	Unknown
lmo1070	lmo1070	Q8Y848	Unknown
lmo2055	lmo2055	Q8Y5K5	Unknown
lmo0800	lmo0800	Q8Y8U3	Unknown
lmo0493	lmo0493	Q8Y9N2	Acylase
lmo2713	lmo2713	Q8Y3W6	Internalin
lmo0227	lmo0227	Q8YAB9	tRNA-dihydrouridine synthase
