# Supplementary material for: Time-Resolved Proteome Analysis of Listeria monocytogenes during Infection Reveals the Role of the AAA+ Chaperone ClpC for Host Cell Adaptation
Source: mSystems. 2021 Aug 3;6(4):e00215-21. doi: 10.1128/mSystems.00215-21 (PMC8407217; doi:10.1128/mSystems.00215-21)
Supplement: TABLE S2 [file msystems.00215-21-st002.pdf]

Identifier			Function	log2 Fold change Δ*clpC*/wt of "new" proteins					
				**Time point (hpi)**					
**Protein name**	**KEGG ID**	**Uniprot ID**		**0,5**	**1**	**1,5**	**2**	**4**	**6**
lmo1961	lmo1961	Q8Y5U4	Ferredoxin--NADP/ Thioredoxin reductase	2,01	2,15	1,88	2,21	1,87	1,9
DltA	lmo0974	Q8Y8D4	Involved in teichoic acid alanylation	-0,52	-0,31	-0,27	-0,2	0,42	0,9
RadA	lmo0233	Q48761	DNA repair protein RadA	0,01	0,45	0,09	-0,1	0,96	0,97
lmo2184	lmo2184	Q7AP55	Heme ABC transporter, heme-binding protein	3,5	2,89	2,72	3,02	2,55	2,61
MotA	lmo0685	Q7AP82	Flagellar motor protein	1,25	1,2	1,1	1,11	1,2	1,18
lmo0119	lmo0119	Q7AP92	Unknown	-0,58	0,14	0,94	0,45	1,75	0,4
LmaA	lmo0118	Q7AP93	Antigen A	-0,12	0,67	1,42	1,13	1,62	1,27
LmaB	lmo0117	Q7AP94	Antigen B	1,16	1	1,21	1,05	1,54	1,37
MurA1	lmo2526	Q8Y4C4	Peptidoglycan precursor biosynthesis protein	0,29	0,68	0,76	0,82	1,41	1,76
MltD	lmo2522	Q8Y4C8	Murein transglycosylase D	2,65	2,23	2,22	3,04	1,88	1,93
FtsX	lmo2506	Q8Y4E1	Cell division protein	1,14	0,86	0,97	0,64	0,51	0,92
lmo2504	lmo2504	Q8Y4E2	Unknown	1,28	1,5	1,28	1,26	0,82	0,87
HupD	lmo2431	Q8Y4K7	Ferrichrome transporter substrate-binding protein	1,37	1,42	1,25	1,09	1,29	1,21
Hbp1	lmo2186	Q8Y585	Hemin/hemoglobin-binding protein 1	3,83	3,54	2,48	3,14	3,12	2,07
lmo2182	lmo2182	Q8Y587	Heme transporter analogous to IsdDEF	3,28	3,08	3,19	3,38	2,91	2,81
SrtB	lmo2181	Q8Y588	Sortase B	3,17	3,04	2,9	3,2	2,47	2,48
FhuD	lmo1959	Q8Y5U6	Ferrichrome-binding periplasmic protein precursor	3,39	5,52	3,69	3,62	3,29	2,59
ReoY	lmo1921	Q8Y5Y2	Adapter protein ReoY	2,83	1,9	4,17	2,18	3,45	2,78
PflA	lmo1917	Q8Y5Y6	Pyruvate-formate-lyase	0,31	0,1	0,19	-0,07	0,66	1,1
CarB	lmo1835	Q8Y665	Carbamoyl-phosphate synthase large chain	0,33	-0,65	-0,82	-1,29	-0,71	-0,27
lmo1244	lmo1244	Q8Y7N0	Phosphoglycerate mutase	0,3	3,75	1,36	0,4	-0,43	1,04
lmo1132	lmo1132	Q8Y7Y7	Unknown	2,32	4,09	4,07	3,31	3,45	3,12
lmo1131	lmo1131	Q8Y7Y8	ABC transporter ATP-binding protein	2,88	2,66	2,22	2,88	2,41	2,59
NifJ	lmo0829	Q8Y8R6	Pyruvate synthase PFOR	0,52	0,89	0,61	0,85	2,02	1,95
lmo0796	lmo0796	Q8Y8U6	Unknown	-1,02	-1,45	0,16	-1,8	-0,61	-0,53
lmo0788	lmo0788	Q8Y8V4	Unknown	0,1	0,37	0,16	0,11	0,82	1,19
lmo0723	lmo0723	Q8Y919	Methyl-accepting chemotaxis protein	1,77	1,48	1,13	0,99	1,23	1,18
FliI	lmo0716	Q8Y926	Flagellum-specific ATP synthase	2,19	1,64	1,36	1,47	2,2	1,44
GmaR	lmo0688	Q8Y949	Flagellar anti-repressor/glycosyltransferase	1,43	1,08	1,37	1,13	1,27	1,6
lmo0675	lmo0675	Q8Y959	Putative flagellar switch protein	2,03	1,36	1,28	1,37	1,21	1,4
lmo0541	lmo0541	Q8Y9I7	ABC transporter substrate-binding protein	2,46	2,88	2,52	2,65	2,42	2,18
lmo0485	lmo0485	Q8Y9P0	Unknown	1,86	2,06	1,72	1,84	1,64	1,42
EfeB	lmo0367	Q8YA00	Ferrous iron transport peroxidase EfeB	4,38	4,66	3,62	4,88	4,91	4,75
EfeO	lmo0366	Q8YA01	Ferrous iron transport periplasmic protein EfeO	3,73	4,16	3,59	3,65	3,65	3,3

Identifier			Function	log2 Fold change clpC/wt of "new" proteins					
				Time point (hpi)					
**Protein name**	**KEGG ID**	**Uniprot ID**		**0,5**	**1**	**1,5**	**2**	**4**	**6**
EfeU	lmo0365	Q8YA02	Ferrous iron transport permease EfeU	4,88	3,65	3,71	3,57	3,53	3,26
lmo0227	lmo0227	Q8YAB9	tRNA-dihydrouridine synthase	0	0,2	0,35	0,34	0,8	1,15
lmo0129	lmo0129	Q8YAJ6	Unknown	1,82	1,15	1,18	1,16	1,36	1,32
lmo0127	lmo0127	Q8YAJ7	Unknown	1,03	1,22	1,39	1,42	2,06	1,94
lmo0125	lmo0125	Q8YAJ9	Unknown	1,87	1,79	1,74	1,67	2,12	2,02
lmo0123	lmo0123	Q8YAK1	Unknown	1,41	1,26	1,23	1,42	1,7	1,68
IsdG	lmo0484	Q92EH3	Heme-degrading monooxygenase	0,96	2,25	1,97	1,8	2,18	1,92
