# Supplementary material for: Time-Resolved Proteome Analysis of Listeria monocytogenes during Infection Reveals the Role of the AAA+ Chaperone ClpC for Host Cell Adaptation
Source: mSystems. 2021 Aug 3;6(4):e00215-21. doi: 10.1128/mSystems.00215-21 (PMC8407217; doi:10.1128/mSystems.00215-21)
Supplement: TABLE S3 [file msystems.00215-21-st003.pdf]

Identifier			Function	log2 Fold change clpC/wt of "old" proteins						
				Time point (hpi)						
Gene name	KEGG	Uniprot		0	0,5	1	1,5	2	4	6
lmo0010	lmo0010	Q8YAV3	Mevalonate kinase	0,39	0,81	0,8	0,88	1	0,92	0,91
AgrA	lmo0051	Q8YAR4	Response regulator	0,08	1,24	1,45	1,51	1,88	1,35	0,9
LmaA	lmo0118	Q7AP93	Antigen A	1,34	0,3	1,2	1,51	1,54	1,14	0,99
lmo0127	lmo0127	Q8YAJ7	unknown function	1,89	1,53	2,41	2,04	2,37	1,83	1,39
lmo0129	lmo0129	Q8YAJ6	unknown function	0,45	1,61	1,17	1,13	1,28	0,45	0,41
lmo0132	lmo0132	Q8YAJ3	Putative IMP dehydrogenase	0,38	0,34	0,64	0,67	0,67	0,36	0,36
hly	lmo0202	P13128	Listeriolysin O precursor	0,21	0,29	0,78	0,59	0,74	0,93	0,43
mpl	lmo0203	P23224	Zinc metalloproteinase precursor	0,16	0,9	0,82	0,78	1,21	0,99	0,88
ActA	lmo0204	P33379	"Actin-assembly inducing protein"	0,14	-0,33	1,02	0,45	1,25	0,24	-1,06
PlcB	lmo0205	P33378	Phospholipase C	0,33	0,28	1,02	0,85	0,94	0,84	0,74
lmo0227	lmo0227	Q8YAB9	tRNA-dihydrouridine synthase	2,01	2,17	2,9	2,83	3,05	2,5	2,54
McsB	lmo0231	Q48759	Protein arginine kinase	0,78	0,71	0,65	1,13	0,59	0,32	1,13
clpC	lmo0232	Q8YAB6	Endopeptidase Clp ATP-binding chain C	-5,37	-3,85	-4,2	-4,29	-3,7	-3,59	-3,46
lmo0279	lmo0279	Q8YA80	Putative ribonucleoside-triphosphate reductase	1,13	2,09	1,8	1,71	1,95	0,67	-0,23
lmo0291	lmo0291	Q8YA68	Unknown	0,82	1,14	1,86	2,1	2,24	2,45	0,57
lmo0360	lmo0360	Q8YA06	Unknown	0,83	0,01	1,24	1	1	0,88	0,52
EfeU	lmo0365	Q8YA02	Ferrous iron transport permease EfeU	0,53	1,29	0,85	0,88	0,98	0,71	0,87
EfeO	lmo0366	Q8YA01	Ferrous iron transport periplasmic protein EfeO	1,08	0,86	1,15	1,29	1,12	1,24	0,91
EfeB	lmo0367	Q8YA00	Ferrous iron transport peroxidase EfeB	1,78	1,82	2,51	2,21	2,56	2,05	1,92
lmo0411	lmo0411	Q8Y9V9	Phosphoenolpyruvate synthase	0,52	1,22	1,5	1,19	1,28	0,76	0,52
lmo0413	lmo0413	Q8Y9V7	Unknown	0,53	0,73	0,74	0,84	1,38	1,15	1
InlB	lmo0434	P0DQD2	Internalin B	0,07	-1,51	0,18	-0,31	0,79	0,04	-0,38
lmo0436	lmo0436	Q8Y9T7	Unknown	0,39	-0,26	0,51	0,28	0,93	0,16	-0,19
lmo0471	lmo0471	Q8Y9Q2	Unknown	0,34	0,44	0,49	0,55	0,84	0,63	0,49
IsdG	lmo0484	Q92EH3	Heme-degrading monooxygenase	0,81	-0,03	0,86	1,05	1,05	1,09	1,12
AroD	lmo0491	Q8Y9N4	3-dehydroquinate dehydratase	0,92	-0,2	0,15	0,72	-1,17	0,68	0,25
lmo0515	lmo0515	Q8Y9L2	UspA-like universal stress protein	0,25	0,59	0,34	0,61	0,83	0,48	0,05
lmo0556	lmo0556	Q8Y9H2	Unknown	0,41	0,93	1,16	1,49	1,44	0,72	1,46
CwhA	lmo0582	P21171	Cell-wall hydrolase A	0,55	0,87	1,31	0,96	1,21	1,2	0,72
lmo0652	lmo0652	Q8Y981	Unknown	-0,09	0,35	0,08	-0,46	0,74	-0,39	NA
lmo0675	lmo0675	Q8Y959	Putative flagellar switch protein	1,39	1,52	1,41	1,34	1,41	1,18	1,18
GmaR	lmo0688	Q8Y949	Flagellar anti-repressor/glycosyltransferase	1,24	1,67	1,51	1,18	1,39	0,98	0,6
CheV	lmo0689	Q8Y948	Chemotaxis protein CheV	1,07	1,42	1,69	1,47	1,62	1,69	1,27
CheA	lmo0692	Q48768	Two-component sensor histidine kinase CheA	1,34	1,73	1,58	1,52	1,78	1,12	0,95

Identifier			Function	log2 Fold change clpC/wt of "old" proteins						
				Time point (hpi)						
Gene name	KEGG	Uniprot		0	0,5	1	1,5	2	4	6
FlgK	lmo0705	Q8Y936	Flagellar hook-associated protein FlgK	1,2	1,06	2,16	1,68	1,99	1,76	0,97
lmo0723	lmo0723	Q8Y919	Methyl-accepting chemotaxis protein	1,47	1,61	2,08	1,35	1,51	1,26	1,17
lmo0764	lmo0764	Q8Y8X8	Lipoate--protein ligase	0,25	0,4	0,3	0,15	1,21	0,33	1,08
lmo0788	lmo0788	Q8Y8V4	Unknown	1,28	1,51	1,63	1,64	1,78	1,57	0,96
lmo0812	lmo0812	Q8Y8T2	Unknown	0,04	0,47	0,13	0,38	1,02	0,19	-1,17
lmo0816	lmo0816	Q8Y8S8	Unknown	0,15	0,04	0,21	0,01	0,85	0,8	-0,11
NifJ	lmo0829	Q8Y8R6	Pyruvate synthase PFOR	2,72	2,43	4,25	3,87	4,03	3,14	2,59
fbp	lmo0830	Q8Y8R5	Fructose-1,6-bisphosphatase class 3	0,42	0,73	0,67	0,65	0,64	0,48	0,66
SigB	lmo0895	Q7AP79	RNA polymerase sigma factor SigB	0,46	0,45	0,75	0,71	0,94	0,65	0,59
lmo0900	lmo0900	Q8Y8K1	Unknown	0,44	-0,27	0,58	0,92	1,1	0,56	-0,04
lmo0904	lmo0904	Q8Y8J7	Unknown	0,26	0,4	0,69	0,69	0,8	0,46	0,52
lmo0965	lmo0965	Q8Y8D9	Unknown	0,03	-1,14	-0,3	0,94	-0,05	0,15	0,1
DltA	lmo0974	Q8Y8D4	Involved in teichoic acid alanylation	1,04	2,13	2,41	2,47	2,56	2,42	2,44
ClpE	lmo0997	Q8Y8B1	ATP-dependent protease	1	0,57	1,19	0,88	0,81	0,81	0,59
lmo1007	lmo1007	Q92D15	Unknown	5,56	2,97	6,65	4,51	4,61	2,86	4,16
lmo1036	lmo1036	Q8Y881	Unknown	0,17	0,37	0,47	0,34	0,67	-0,04	0,01
lmo1113	lmo1113	Q8Y806	Unknown	0,8	1,12	1,58	1,28	1,52	1,49	1,17
lmo1118	lmo1118	Q8Y801	Unknown	0,73	0,75	1,32	0,93	1,47	1,33	1,02
lmo1132	lmo1132	Q8Y7Y7	Unknown	0,42	0,71	0,59	0,63	0,79	0,48	0,67
ProB	lmo1260	Q93Q56	Glutamate 5-kinase	0,07	0,05	0,31	0,27	0,65	0,3	0,28
LuxS	lmo1288	Q8Y7I9	S-ribosylhomocysteine lyase	0,62	0,83	0,24	0,61	0,72	-0,09	-0,32
lmo1309	lmo1309	Q8Y7H1	Unknown	1,02	1,38	2,49	2,14	2,78	1,89	1,3
lmo1384	lmo1384	Q8Y7A4	Unknown	0,49	1,39	1,66	2,02	1,63	1,11	0,75
LadR	lmo1408	Q7AP72	PadR-related transcriptional regulator	0,71	0,36	0,99	0,27	0,88	0,32	0,22
lmo1423	lmo1423	Q8Y774	Unknown	0,24	0,3	0,69	0,48	1,14	0,85	0,68
LytB / IspH	lmo1451	P58676	Diphosphate reductase	0,36	1,27	1,06	1,4	1,18	0,86	3,43
lmo1521	lmo1521	Q8Y707	Unknown	0,21	0,47	0,72	0,56	0,78	0,64	0,59
HemA	lmo1557	Q8Y6X4	Glutamyl-tRNA reductase	1,35	2,27	2,72	2,75	3,01	2,66	2,91
LDH	lmo1667	Q8Y6L7	L-lactate dehydrogenases	0,46	0,52	0,77	0,95	0,9	0,82	1,11
lmo1678	lmo1678	Q8Y6K6	Unknown	0,3	1,18	0,88	0,96	0,88	0,04	0,46
lmo1679	lmo1679	Q8Y6K5	Unknown	0,79	0,88	0,97	1,44	1,05	0,82	1,36
lmo1699	lmo1699	Q8Y6I5	Chemotaxis protein	1,65	1,58	1,62	1,54	2,02	1,54	0,97
MreBH	lmo1713	Q8Y6H3	Component of the Rod- complex	0,55	0,66	0,68	1,05	0,59	0,75	0,66
lmo1778	lmo1778	Q8Y6B3	Unknown	-0,02	0,7	-0,06	0,37	0,72	0,39	0,5

Identifier			Function	log2 Fold change clpC/wt of "old" proteins						
				Time point (hpi)						
Gene name	KEGG	Uniprot		0	0,5	1	1,5	2	4	6
lmo1867	lmo1867	Q8Y633	Pyruvate phosphate dikinase	0,28	0,77	0,65	0,59	0,79	0,56	0,49
RecU	lmo1891	Q8Y611	Holliday junction resolvase RecU	0,93	0,77	1,62	1,04	1,9	1,72	1,3
PbpA1	lmo1892	Q8Y610	Unknown	0,42	0,42	0,59	-0,08	0,71	0,25	-0,21
PflA	lmo1917	Q8Y5Y6	Pyruvate-formate-lyase	0,78	1,09	1,01	1,09	1,29	1,24	1,41
ReoY	lmo1921	Q8Y5Y2	Adapter protein ReoY	2,85	2,04	2,74	3,09	2,63	1,67	1,12
XerD	lmo1955	Q8Y5V0	Tyrosine recombinase	0,4	0,58	0,77	0,7	1,01	0,67	0,84
FhuD	lmo1959	Q8Y5U6	Ferrichrome-binding protein precursor	0,98	0,8	0,97	1,05	0,88	1,01	0,52
FhuC	lmo1960	Q8Y5U5	Ferrichrome transport ATP-binding protein	0,86	0,73	0,86	0,67	1,03	0,87	0,99
lmo1961	lmo1961	Q8Y5U4	Ferredoxin--NADP/ Thioredoxin reductase	0,36	1,05	0,73	0,71	0,91	0,94	0,69
IlvA	lmo1991	Q8Y5R5	L-threonine dehydratase	0,54	0,7	0,76	0,93	0,97	0,74	0,85
alsS	lmo2006	Q8Y5Q0	Glucose-6-phosphate 1-dehydrogenase	0,09	0,59	0,76	0,57	0,71	0,51	0,61
lmo2060	lmo2060	Q8Y5K0	Unknown	-0,36	-0,37	0,28	-0,3	1,01	-0,12	0,04
lmo2128	lmo2128	Q8Y5D7	Unknown	0,29	1,12	0,73	0,78	0,91	0,85	0,81
lmo2132	lmo2132	Q8Y5D3	Unknown	0,63	0,68	0,99	0,77	1,05	1,23	1,02
lmo2143	lmo2143	Q8Y5C2	Unknown	0,56	0,84	0,98	0,64	1,12	0,84	1,17
lmo2156	lmo2156	Q8Y5B1	Unknown	0,76	0,99	1,57	1,34	2,27	1,09	1,08
lmo2182	lmo2182	Q8Y587	Heme transporter analogous to IsdDEF	0,71	0,54	0,81	1,24	0,85	0,97	0,77
lmo2184	lmo2184	Q7AP55	Heme ABC transporter, heme-binding protein	0,78	0,86	1,09	0,95	1,03	0,95	0,8
Hbp2 /SvpA	lmo2185	Q7AP54	Hemin/hemoglobin-binding protein 2	1,15	0,58	0,65	0,7	0,44	0,32	0,15
Hbp1	lmo2186	Q8Y585	Hemin/hemoglobin-binding protein 1	1,15	0,8	0,91	0,9	0,74	0,96	0,6
MecA	lmo2190	Q9RGW9	Adapter protein MecA	2,18	2,83	3,07	3,74	3,62	2,03	2,29
ClpB	lmo2206	Q8Y570	Chaperone proteins	0,42	0,72	0,69	0,62	0,77	0,42	0,34
lmo2209	lmo2209	Q8Y567	Unknown	0,22	0,44	0,6	0,82	1,07	0,48	0,33
AddA	lmo2267	Q8Y511	ATP-dependent helicase/nuclease subunit A	0,39	0,56	0,59	0,53	0,64	0,27	0,17
lmo2296	lmo2296	Q8Y4Y3	Unknown	0,71	0,85	0,73	0,76	1	1,1	1,02
int	lmo2332	Q8Y4U8	Putative integrase [Bacteriophage A118]	0,21	0,58	0,52	0,41	0,72	0,44	0,55
lmo2343	lmo2343	Q8Y4T9	Unknown	1,17	0,75	1,68	1,74	1,51	1,69	1,99
lmo2345	lmo2345	Q8Y4T7	Unknown	0,42	1,2	0,93	1	1,15	0,9	1,3
lmo2351	lmo2351	Q8Y4T1	Unknown	0,45	0,69	0,88	1,26	1,31	1,37	1,41
lmo2368	lmo2368	Q8Y4R6	Unknown	0,22	0,52	0,69	0,75	0,95	1,3	0,44
GadD3	lmo2434	Q8Y4K4	Glutamate decarboxylases	0,21	0,58	0,6	0,54	0,61	0,29	0,31
lmo2439	lmo2439	Q8Y4J9	Unknown	-0,23	0,8	0,87	0,96	1,22	0,38	0,34
WhiA	lmo2472	Q8Y4H0	Probable cell division protein WhiA	0,21	0,64	0,65	0,45	0,7	0,65	0,6
lmo2504	lmo2504	Q8Y4E2	Unknown	0,71	0,66	2,25	1,64	1,53	0,86	-0,43

Identifier			Function	log2 Fold change clpC/wt of "old" proteins						
				Time point (hpi)						
Gene name	KEGG	Uniprot		0	0,5	1	1,5	2	4	6
MltD	lmo2522	Q8Y4C8	Murein transglycosylase D	1,98	0,28	0,45	-0,16	-0,24	-0,02	1,11
MurA1	lmo2526	Q8Y4C4	Peptidoglycan precursor biosynthesis protein	1,52	2,19	2,61	2,55	2,71	2,64	2,28
HrtB	lmo2581	Q8Y474	ABC transport system permease protein	1,1	1,59	1,31	1,37	1,47	1,47	1,17
lmo2643	lmo2643	Q8Y431	Unknown	0,2	0,74	0,61	0,61	0,77	0,52	0,65
lmo2683	lmo2683	Q8Y3Z5	Unknown	-0,32	-1,05	-1,36	-0,58	-1,59	-0,65	-1,68
lmo2829	lmo2829	Q8Y3K6	Unknown	-0,63	-1,63	-1,32	0,05	-1,12	-0,51	-0,82
lmo2857	lmo2857	Q8Y3I0	Unknown	-0,55	-0,14	0,63	-1,16	-0,28	-0,61	0,31
