# Supplementary material for: Time-Resolved Proteome Analysis of Listeria monocytogenes during Infection Reveals the Role of the AAA+ Chaperone ClpC for Host Cell Adaptation
Source: mSystems. 2021 Aug 3;6(4):e00215-21. doi: 10.1128/mSystems.00215-21 (PMC8407217; doi:10.1128/mSystems.00215-21)
Supplement: TABLE S4 [file msystems.00215-21-st004.pdf]

Identifier			Function	log2 Half-life clpC/wt
Protein name	KEGG ID	Uniprot ID		
GyrA	lmo0007	Q8YAV6	DNA gyrase subunit A	2,31
lmo0009	lmo0009	Q8YAV4	Unknown	2,69
lmo0011	lmo0011	Q8YAV2	Putative mevalonate kinase	2,63
lmo0018	lmo0018	Q8YAU5	Beta-glucosidase	2,82
MccF	lmo0028	Q8YAT5	Microcin C7 self-immunity protein MccF	2,41
lmo0132	lmo0132	Q8YAJ3	Putative IMP dehydrogenase	2,71
HolB	lmo0162	Q8YAG4	DNA polymerase III subunit delta	2,24
lmo0170	lmo0170	Q8YAF6	Unknown	3,31
MetG	lmo0177	Q8YAF2	Methionine aminoacyl-tRNA ligase	2,06
lmo0184	lmo0184	Q8YAE6	Oligo-1,6-glucosidase	3,62
lmo0241	lmo0241	Q92F34	Unknown	2,63
RpoB	lmo0258	Q9RLT9	DNA-dependent RNA polymerase RpoB	2,29
lmo0271	lmo0271	Q8YA88	6-phospho-beta-glucosidase	2,34
lmo0277	lmo0277	Q8YA82	Oxidoreductase	2,83
lmo0342	lmo0342	Q8YA23	Transketolase	2,59
lmo0352	lmo0352	Q8YA14	Unknown	2,38
lmo0356	lmo0356	Q8YA10	Oxidoreductase	3,59
RocG	lmo0560	Q8Y9G8	Glutamate dehydrogenase	2,65
HisD	lmo0567	Q8Y9G1	Histidinol dehydrogenase	-3,07
SecA2	lmo0583	P0DJP3	Part of Sec translocase system	2,89
HflXr	lmo0762	Q8Y8Y0	GTPase	-2,47
lmo0845	lmo0845	Q8Y8Q1	Unknown	2,32
lmo0900	lmo0900	Q8Y8K1	Unknown	-2,39
lmo0906	lmo0906	Q8Y8J5	Putative glutathione reductase	2,52
GabD	lmo0913	Q8Y8I9	Succinic semialdehyde dehydrogenase	2,83
DltA	lmo0974	Q8Y8D4	Involved in teichoic acid alanylation	2,81
PtsI	lmo1003	O31149	Phosphoenolpyruvate-protein phosphotransferase	2,58
rnj	lmo1027	Q92CZ5	RNase J	2,21
lmo1057	lmo1057	Q8Y860	Oxidoreductase	2,1
lmo1085	lmo1085	Q8Y833	Unknown	2,56
lmo1223	lmo1223	Q8Y7Q0	Unknown	2,51
lmo1235	lmo1235	Q8Y7N9	Putative aspartokinase	2,18
lmo1241	lmo1241	Q8Y7N3	Unknown	2,1
hfq	lmo1295	Q92C58	RNA chaperone	-2,46
dxr	lmo1317	Q8Y7G4	1-deoxy-D-xylulose 5-phosphate reductoisomerase	2,63
ProS	lmo1319	Q8Y7G2	Prolyl aminoacyl-tRNA ligase	3,53

Identifier			Function	log2 Half-life clpC/wt
Protein name	KEGG ID	Uniprot ID		
lmo1351	lmo1351	Q8Y7D2	Unknown	3,02
RecN	lmo1368	Q8Y7B8	Involved in DNA repair	2,14
lmo1371	lmo1371	Q8Y7B5	Dihydrolipoyl dehydrogenase	2,5
lmo1376	lmo1376	Q8Y7B0	Synthesis of ribulose 5-phosphate from glucose 6-phosphate	3,75
RodZ	lmo1395	Q8Y795	Component of the Rod- complex	2,15
CinA	lmo1397	Q8Y793	Putatively involved in fatty acd biosynthesis	2,54
lmo1433	lmo1433	Q8Y768	Putative glutathione reductase	2,42
asd	lmo1437	Q8Y764	Aspartate-semialdehyde dehydrogenase	3,16
CshB	lmo1450	Q8Y755	DEAD-Box RNA helicase	2,95
GlyS	lmo1458	Q8Y754	Glycine aminoacyl-tRNA ligase	2,3
lmo1467	lmo1467	Q7AP63	Unknown	2,45
RpsU	lmo1469	P0DJP1	30S ribosomal protein S21	3,6
RsmE	lmo1470	Q7AP62	putative methyltransferase	2,62
LepA	lmo1479	Q8Y742	Elongation factor 4	2,42
lmo1495	lmo1495	Q8Y728	Unknown	3,4
lmo1514	lmo1514	Q8Y712	Unknown	2,25
HisS	lmo1520	Q8Y708	Histidine aminoacyl-tRNA ligase	2,11
tgt	lmo1530	Q8Y700	Putative queuine tRNA-ribosyltransferase	2,49
Ldh2	lmo1534	Q8Y6Z6	L-lactate dehydrogenase	2,1
GlpK	lmo1538	Q8Y6Z2	Glycerol kinase	2,45
RplU	lmo1542	Q8Y6Y9	50S ribosomal protein L21	2,34
valS	lmo1552	Q8Y6X9	Valine aminoacyl-tRNA ligase	2,4
HemC	lmo1556	Q8Y6X5	Conversion of ALA to coproporphyrinogen-III	2,04
HemA	lmo1557	Q8Y6X4	Glutamyl-tRNA reductase	3,46
EngB	lmo1558	Q8Y6X3	GTP-binding protein	2,54
PykA	lmo1570	Q8Y6W1	Pyruvate kinase	2,58
TyrS	lmo1598	Q8Y6T4	Tyrosine aminoacyl-tRNA ligase	2,8
AroA	lmo1600	Q8Y6T2	DAHP-synthase	2,3
lmo1642	lmo1642	Q8Y6P2	Unknown	2,43
lmo1645	lmo1645	Q8Y6N9	Unknown	2,5
RpsB	lmo1658	Q8Y6M6	30S ribosomal protein S2	2,08
LeuS	lmo1660	Q8Y6M4	Leucine aminoacyl-tRNA ligase	2,4
AnsB	lmo1663	Q8Y6M1	Aspartase	2,79
MenF	lmo1676	Q8Y6K8	Isochorismate synthase	2,66
lmo1680	lmo1680	Q8Y6K4	Cystathionine gamma-synthase	3,59
lmo1722	lmo1722	Q8Y6G5	DEAD-Box RNA helicase	2,6

Identifier			Function	log2 Half-life clpC/wt
Protein name	KEGG ID	Uniprot ID		
lmo1744	lmo1744	Q8Y6E3	Unknown	3,36
GatB	lmo1754	Q8Y6D3	Aspartyl/glutamyl-tRNA amidotransferase subunit B	2,23
PurH	lmo1765	Q8Y6C5	IMP cyclohydrolase	2,27
PepT	lmo1780	Q8Y6B1	Aminopeptidase T	2,62
RplT	lmo1783	P66103	50S ribosomal protein L20	2,63
RplS	lmo1787	O53083	50S ribosomal protein L19	2,65
lmo1807	lmo1807	Q8Y690	Putatively involved in fatty acd biosynthesis	2,13
RecG	lmo1811	Q8Y686	DNA helicase	2,58
PriA	lmo1824	Q8Y675	Primosome assembly protein PriA	2,03
PyrB	lmo1838	Q8Y662	Aspartate carbamoyltransferase	2,29
lmo1858	lmo1858	Q8Y642	Unknown	2,23
lmo1867	lmo1867	Q8Y633	Pyruvate phosphate dikinase	2,14
AnsS	lmo1896	P58695	Asparagine aminoacyl-tRNA ligase	2,28
AspB	lmo1897	Q8Y606	Aspartate aminotransaminase	2,13
cca	lmo1905	Q8Y5Z8	Putative nucleotidyltransferase	2,41
lmo1910	lmo1910	Q8Y5Z3	Unknown	2,04
MaeA	lmo1915	Q8Y5Y8	Malic enzyme	2,48
AroE	lmo1923	Q8Y5Y0	3-phosphoshikimate 1-carboxyvinyltransferase	2,6
lmo1930	lmo1930	Q8Y5X3	Unknown	2,11
lmo1949	lmo1949	Q8Y5V6	Putative rRNA pseudouridine synthase	2,47
lmo1961	lmo1961	Q8Y5U4	Ferredoxin--NADP reductase 1 / Thioredoxin reductase	2,39
rnz	lmo1977	Q8Y5S8	RNase Z	2,72
zwf	lmo1978	Q8Y5S7	Glucose-6-phosphate dehydrogenase	2,83
LeuA	lmo1987	Q8Y5R9	Isopropylmalate synthase	2,46
FtsA	lmo2033	Q8Y5M4	Cell division protein FtsA	2,27
MurD	lmo2036	Q8Y5M1	UDP-N-acetylmuramoyl-L-alanyl-D-glutamate synthetase	2,21
MurE	lmo2038	Q8Y5L9	UDP-N-acetylmuramoylalanyl-D-glutamate--2,6-diaminopimelate ligase	2,35
lmo2050	lmo2050	Q8Y5K9	Unknown	2,62
rex	lmo2072	P60384	Redox-sensing transcriptional repressor	2,39
lmo2077	lmo2077	Q8Y5I5	Unknown	3,14
DacA	lmo2120	Q8Y5E4	Diadenylate cyclase	2,03
SepA	lmo2157	Q8Y5B0	Similar to ABC transporter permease	2,52
lmo2188	lmo2188	Q8Y583	Acetolactate synthase	2,44
OppA	lmo2195	Q8Y579	Extracellular solute binding protein of Opp transporter	2,9
FabF	lmo2201	Q8Y574	Acyl chain elongation	2,47
ClpB	lmo2206	Q8Y570	Chaperone proteins	2,44

Identifier			Function	log2 Half-life clpC/wt
Protein name	KEGG ID		Uniprot ID	
PepC	lmo2338	O69192	Aminopeptidase C	2,06
lmo2345	lmo2345	Q8Y4T7	Unknown	2,48
lmo2354	lmo2354	Q8Y4S8	Unknown	2,21
pgi	lmo2367	Q8Y4R7	Glucose-6-phosphate isomerase	2,39
SufD	lmo2414	Q8Y4M3	Component of the Suf system	2,34
GadD3	lmo2434	Q8Y4K4	Glutamate decarboxylases	3,37
SmpB	lmo2448	P66860	Involved in the addition SsrA tag	2,58
rnr	lmo2449	Q8Y4J0	RNaseR	2,66
GAPDH	lmo2459	Q8Y4I1	Glyceraldehyde-3-phosphate dehydrogenase	2,59
TrxB	lmo2478	O32823	Probable thioredoxin reductase	3,21
HprK	lmo2483	Q8Y4G1	Phosphatase	2,68
rho	lmo2551	Q8Y4A3	Helicase	2,22
PyrG	lmo2559	Q8Y495	Converts carbamoyl phosphate and aspartate to N-carbamoylaspartate	2,24
ArgS	lmo2561	Q8Y493	Arginine aminoacyl-tRNA ligase	2,39
RpsI	lmo2596	Q8Y459	30S ribosomal protein S9	2,57
RplM	lmo2597	Q8Y458	50S ribosomal protein L13	3,89
RplR	lmo2616	Q8Y445	50S ribosomal protein L18	2,55
RplF	lmo2617	Q8Y444	50S ribosomal protein L6	2,37
RplN	lmo2622	Q927L7	50S ribosomal protein L14	2,66
RpsC	lmo2626	P66548	30S ribosomal protein S3	2,11
RplB	lmo2629	P60426	50S ribosomal protein L2	3,47
RplD	lmo2631	P61055	50S ribosomal protein L4	2,18
RplC	lmo2632	Q8Y440	50S ribosomal protein L3	3,08
RpsG	lmo2655	P66611	30S ribosomal protein S7	3,15
RecQ	lmo2757	Q8Y3S4	ATP-dependent DNA helicase RecQ	-2,40
KatG	lmo2785	Q8Y3P9	Catalase	2,43
GidA/MnmG	lmo2810	Q8Y3M5	Mediates a tRNA wobble uridine modification	2,76
lmo2815	lmo2815	Q8Y3M0	Putatively involved in fatty acid biosynthesis	2,4
