# Supplementary material for: Time-Resolved Proteome Analysis of Listeria monocytogenes during Infection Reveals the Role of the AAA+ Chaperone ClpC for Host Cell Adaptation
Source: mSystems. 2021 Aug 3;6(4):e00215-21. doi: 10.1128/mSystems.00215-21 (PMC8407217; doi:10.1128/mSystems.00215-21)
Supplement: TABLE S5 [file msystems.00215-21-st005.pdf]

Primer code	F, forward primer; R, reverse primer	Use	Sequence 5´-3′
**OLEC8511**	R	Isolation of N-terminal-flank (855 bp) sequence uptream of *lysA* with pMAD overhangs	GTGGGATTGGCGTGACGTTT*tcattatagtagggcgggacagatatg*
**OLEC8510**	F		GCCATGGTACCCGGGAGCTCGAATTC*caaactctctaatacgcctaattgttcttc*
**OLEC8509**	R	Isolation of C-terminal-flank (857 bp) sequence downstream of *lysA* with pMAD overhangs	GCGTCGGGCGATATCGGATCC*gtgagttagaaaataaagaagttgtggcaatgc*
**OLEC8508**	F		CATATCTGTCCCGCCCTACTATAATGA*aaacgtcacgccaatcccac*
**OLEC8803**	R	pMAD sequencing	CTTAAAATAATTGGTAGCTAATCTCAG
**OLEC7174**	F		GCAACGCGGGCATCCCGATG
**OLEC7175**	F		CCCAATATAATCATTTATCAACTCTTTTACACTTAAATTTCC
**OLEC8848**	F	Isolation of nesting fragment from the genome containing the *lysA* deletion	*gtcagcactttcgttatatcg*
**OLEC8849**	R		*gtgcttgcggatgaagtaaac*
**OLEC8509**	R	Sequencing of *lysA* deletion in the genome	GCGTCGGGCGATATCGGATCC*gtgagttagaaaataaagaagttgtggcaatgc*
**OLEC8802**	F		*cttgcattgtatgaacaaattccatg*
**OLEC8510**	F		GCCATGGTACCCGGGAGCTCGAATTC*caaactctctaatacgcctaattgttcttc*
**OLEC3681**	R	Isolation of nesting fragment from the genome containing the *clpC* deletion	*gcggatgctatttcgg*
**OLEC3680**	F		*caagtcctacatctcg*
**OLEC9405**	R	Sequencing of *clpC* deletion in the genome	*catatccacatgcctgacacac*
