# Supplementary material for: Time-Resolved Proteome Analysis of Listeria monocytogenes during Infection Reveals the Role of the AAA+ Chaperone ClpC for Host Cell Adaptation
Source: mSystems. 2021 Aug 3;6(4):e00215-21. doi: 10.1128/mSystems.00215-21 (PMC8407217; doi:10.1128/mSystems.00215-21)
Supplement: TABLE S6 [file msystems.00215-21-st006.pdf]

Strain name	Characteristics	Source
**Parental strain (EGD-e)** EC2883	Wild type EGD-e	Pascale Cossart Lab<br>RefSeq accession number NC_003210
**wt (∆*lysA*)** EC2886	EC2883ΔlysA (Δl*mo1952)*	This study
***∆clpC* (∆*lysA*∆*clpC*)** EC2887	EC2886∆*clpC* (Δ*lmo0232)*	This study
